# Novel semi-automated fluorescence microscope imaging algorithm for monitoring IgG aggregates in serum

**DOI:** 10.1038/s41598-021-90623-7

**Published:** 2021-05-31

**Authors:** Shravan Sreenivasan, Deepak Sonawat, Shyamapada Mandal, Kedar Khare, Anurag S. Rathore

**Affiliations:** 1 Department of Chemical Engineering, DBT Center of Excellence for Biopharmaceutical Technology, Indian Institute of Technology Delhi, Hauz Khas, New Delhi 110016 India; 2 Department of Physics, Indian Institute of Technology Delhi, Hauz Khas, 110016 India

**Keywords:** Biological techniques, Mathematics and computing

## Abstract

Analysis of therapeutic IgG aggregates in serum is a potential area of investigation as it can give deeper insights about the function, immunogenic issues and protein interaction associated with the aggregates. To overcome various complexities associated with the existing analytical techniques for analyzing aggregates in serum, a novel florescence microscopy-based image processing approach was developed. The monoclonal antibody (mAb) was tagged with a fluorescent dye, fluorescein isothiocyanate (FITC). Aggregates, generated by stirring, were spiked into serum and images were captured at various time points. After denoising, thresholding by weighted median, 1D Otsu, and 2D Otsu was attempted and a modified 2D Otsu, a new mode of thresholding, was developed. This thresholding method was found to be highly effective in removing noises and retaining analyte sizes. Out of 0–255, the optimized threshold value obtained for the images discussed in modified 2D Otsu was 9 while 2D Otsu’s overestimated values were 38 and 48. Other morphological operations were applied after thresholding and the area, perimeter, circularity, and radii of the aggregates in these images were calculated. The proposed algorithm offers an approach for analysis of aggregates in serum that is simpler to implement and is complementary to existing approaches.

## Introduction

Therapeutic monoclonal antibodies (mAbs) form aggregates during various stages of production, transportation, and storage. These aggregates can result in altered biological activity and adverse immune responses^1,2^. Analysis of sub-visible (0.1–50 µm) and higher sized (> 50 µm) aggregates is popular due to the adverse immune responses they may cause^3,4^.

The aggregates of therapeutic IgG have altered properties as compared to the monomer^1,5–8^. When aggregates are introduced into the blood, various undesired outcomes such as blockage of blood capillaries, change in efficacy, clearance, and adverse immune reactions can occur^3,6,9^. Aggregate size and its nature can further change in the blood due to its interaction with other aggregates and blood proteins^3,8–10^. Hence, understanding the behavior of therapeutic IgG aggregates in biological fluids such as plasma and serum is of great interest^3,6,9,10^. Serum has various proteins already present in it^3,8^. As a result, characterization of aggregates in serum is difficult, if not impossible, via the existing tools such as size exclusion chromatography (SEC), nanoparticle tracking analysis (NTA), dynamic light scattering (DLS), asymmetrical field-flow fractionation (FFF), light obscuration (LO), particle counting, analytical ultracentrifugation (AUC) and flow-based imaging techniques^5–7^. As a result, the knowledge of how aggregates evolve once in blood or serum is quite limited^3,6,10,11^. Although specialized techniques such as fluorescence single particle tracking (fSPT), confocal laser scanning microscopy (CLSM), AUC with fluorescence detection system (FDS), optical microscopy, and flow cytometry (FCM) have been reported^3,6,9,11^, they suffer from drawbacks such as limited size range, complex optimization of settings, sample dilution in instrument fluid and application of forces such as centrifugation^3,4,6,9,11,12^. In addition to it, these instruments require very high investment and trained users^4,12^.

Fluorescence microscope, a type of optical microscope, enables us to selectively measure aggregate size and morphology in cells and other biological fluids^4,11^. It works on the principle of fluorescence where the analyte is irradiated using light from an excitation source. The sample then emits light of higher wavelength, which is selectively detected. Other than tagging the sample with fluorescent dye, this technique does not require any complex sample preparation steps. A simple widefield fluorescence microscope is cheaper, easier to maintain, use and house. Wider illumination areas combined with lesser instrumental and operational complications are more of its distinctive features. Images in a fluorescence microscope can be acquired with reduced exposure times, which can further enable recording of fast-moving particles^4,11,12^.

Use of microscopy-based methods have a general disadvantage that only a small fraction of the sample can be analyzed at a time. Hence the output from a single image might not be a true representation of the entire sample. So, multiple images of a particular sample containing aggregates must be obtained. Fluorescence microscope has an additional drawback that its images are corrupted by noise and blurred particles from out of focal plane. An image of aggregate in a mAb sample captured by this technique is shown in Supplementary Fig. 1. The noises, which results in images with very low signal-by-noise ratio (SNR), are due to the stray light from the background, inaccuracy in the detection and emission of light (Poisson noise), and incorrect quantification by the detector (Gaussian noise)^13^. The experimental parameters and auto-fluorescence from untagged regions also contribute to noise. The noises are removed by using various image processing techniques which include different de-noising and thresholding steps^14,15^. A lot of algorithms for noise removal have been proposed in the literature, but most of them suffer from major drawbacks such as blurring of target aggregates, false detection of noise, improper local illumination as target particles, and false removal of low intensity aggregates as noise^13,16,17^.

**Figure 1:**
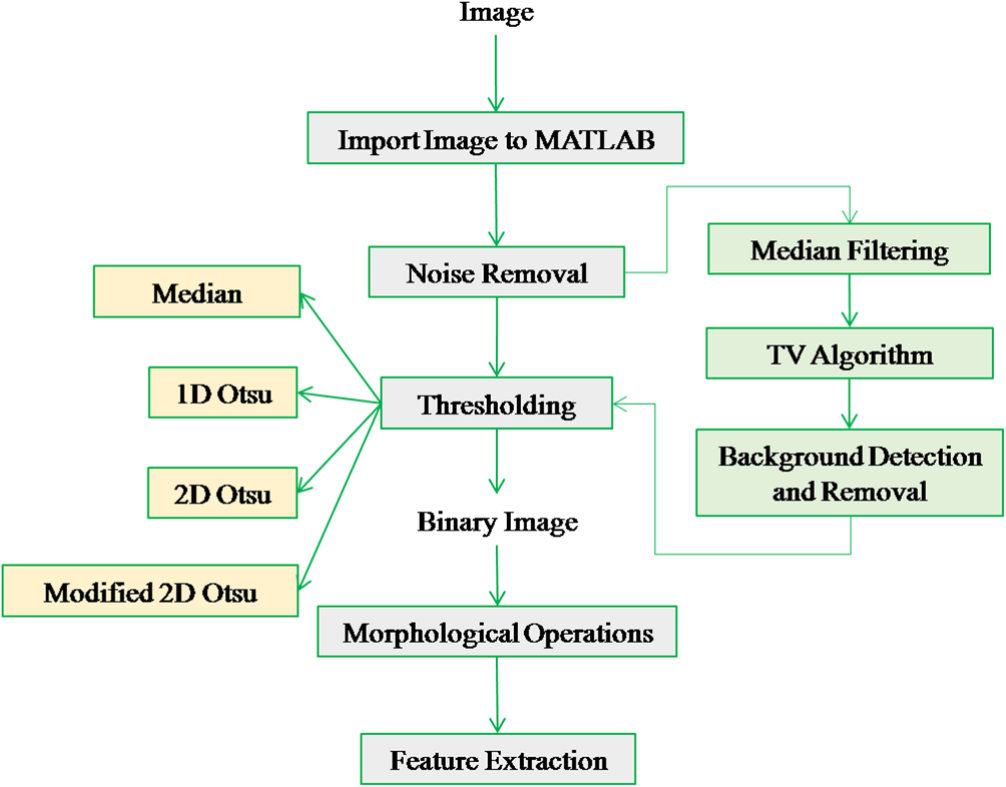
The overall procedure showing the different steps for processing the fluorescence microscope images.

In this work, a novel image processing algorithmic sequence is proposed for the analysis of samples containing sub-visible and visible aggregates in serum using fluorescence microscopy. The series of specific denoising steps address the different noises identified for the images. The different steps used in image processing being mentioned henceforth have been previously published elsewhere^18–23^. But its application towards analysis of IgG aggregates in serum has not yet been reported. The aggregate was made by introducing mechanical shear by stirring the mAb tagged with a fluorescent dye, fluorescein isothiocyanate (FITC). These aggregates were introduced into serum and microscope images were captured at varying time points. Processing of these images was performed using the inbuilt ‘image processing toolbox’ in MATLAB. The proposed algorithm successfully addressed various noise reduction methods along with different modes of thresholding. A comparison among the addressed methodologies has also been presented.

## Approach to image analysis

The complete procedure of analyzing images is shown in Fig. 1. The images are imported into MATLAB in the form of a 3-dimensional (3D) matrix. The noises are removed using median filtering followed by total variation (TV) de-noising algorithm. The illumination noise is further reduced by separating and removing the background using fast Fourier transformation. The processed images are then converted into binary images by thresholding. Various thresholding methods such as weighted median, 1-dimensional (1D) Otsu and 2-dimensional (2D) Otsu thresholding are applied. A new modified 2D Otsu thresholding approach has been proposed to address the low image to background ratio problem in 2D Otsu approach and increase in size of aggregates in modified median thresholding approach. Morphological operations are then performed to remove unwanted pixels. The particles are labeled and features such as size distribution and circularity are calculated.

### Importing image as readable data

Fluorescence microscope images of the mAb aggregates are imported into MATLAB as 3D matrix of dimension J × K × 3. The third dimension of the matrix is designated to each colour (channel) i.e. Red (R), Green (G) and Blue (B) channels. J × K is the number of pixels in the image, where J points to the row and K to the column. Value in pixels of each channel ranges from 0 to 255, where 0 indicates absence of transmission of colour intensity and 255 indicates complete presence of transmission of that colour intensity. FITC is a derivative of fluorescein where an isothiocyanate reactive group (–N=C=S) replaces an H atom at the bottom ring of fluorescein, imparting green colour to the analyte^18^. Hence, the contributions of blue and red channels were neglected. Only green channel data $g\left( {x,y} \right)$ is taken forward for processing.

### De-noising

The analyzed image is composed of true signals along with different types of noises whose removal requires multiple steps.

#### Median filtering

**Figure 2:**
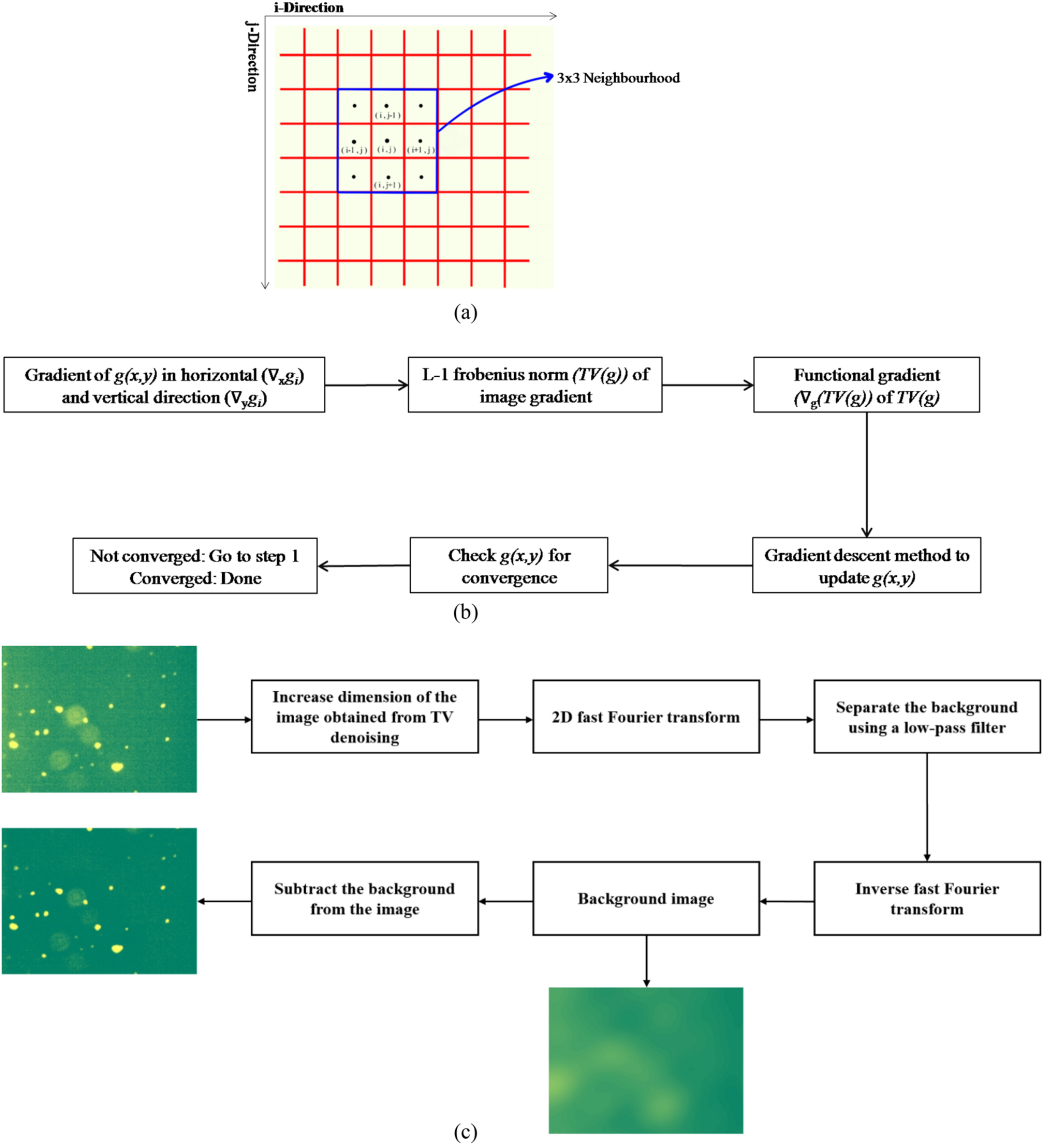
Various operations involved in de-noising steps where (**a**) is the median filtering, (**b**) is the steps involved in TV algorithm and (**c**) summarizes the process of background subtraction.

Median filtering is a common noise reduction method used in image processing. In this method the value of $\left( {i,j} \right)^{th}$ pixel is replaced with the median of the chosen N × N neighbourhood (Fig. 2a). The neighbourhood, N, is user defined. This method is effective on images with random noise or long-tailed histograms of images^19^.

#### TV denoising algorithm and background normalization

Total variation $\left( {TV\left( {g\left( {x,y} \right)} \right)} \right)$ is the L-1 norm of the gradient of image (Eq. 1) in horizontal $\left( {\nabla_{x} g} \right)$ and vertical $\left( {\nabla_{y} g} \right)$ direction. Total variation captures the local fluctuations (noise) in the image. TV reduction is achieved by recursive gradient descent method (Eq. 2) (Gaur et al., 2015). Convergence criteria (Eq. 4) are defined as the relative change in the image from $i^{th}$ to $\left( {i + 1} \right)^{th}$ iteration. Tolerance level for convergence is user defined. The approach used in reducing total variance is shown in Fig. 2b.

1 $$TV\left( g \right) = \left| {\left| {\nabla g} \right|} \right|_{1} = \mathop \sum \limits_{i = allpixels} \sqrt {\left| {\nabla_{x} g_{i} } \right|^{2} + \left| {\nabla_{y} g_{i} } \right|^{2} } ,$$

2 $$g^{{\left( {n + 1} \right)}} = g^{\left( n \right)} - \tau \left[ {\nabla_{g} TV\left( g \right)} \right]_{{g = g^{\left( n \right)} }} ,$$

3 $$\left[ {\nabla _{g} TV\left( g \right)} \right]_{{g = g^{{\left( n \right)}} }} = - \left[ {\nabla \cdot \left[ {\frac{{\nabla g}}{{\sqrt {\left| {\nabla g} \right|^{2} + \varepsilon ^{2} } }}} \right]} \right]_{{g = g^{{\left( n \right)}} }} ,$$

4 $$E_{n} = \left[ {\frac{{\left| {\left| {g^{{\left( {n + 1} \right)}} \left( {x,y} \right) - g^{\left( n \right)} \left( {x,y} \right)} \right|} \right|}}{{\left| {\left| {g^{\left( n \right)} \left( {x,y} \right)} \right|} \right|}}} \right] < tolerance.$$

Here, || … || represents the L-2 norm and τ is the step size determined by line search. Image obtained after TV denoising method is used to normalize the background. A low pass filtered image is obtained by applying the fast Fourier transform. The TV denoised image is divided pixel-by-pixel by the low-pass filtered image. The image obtained after this operation has normalized background which helps in uniform thresholding on the image. The steps for normalizing background are shown in Fig. 2c.

### Thresholding

**Figure 3:**
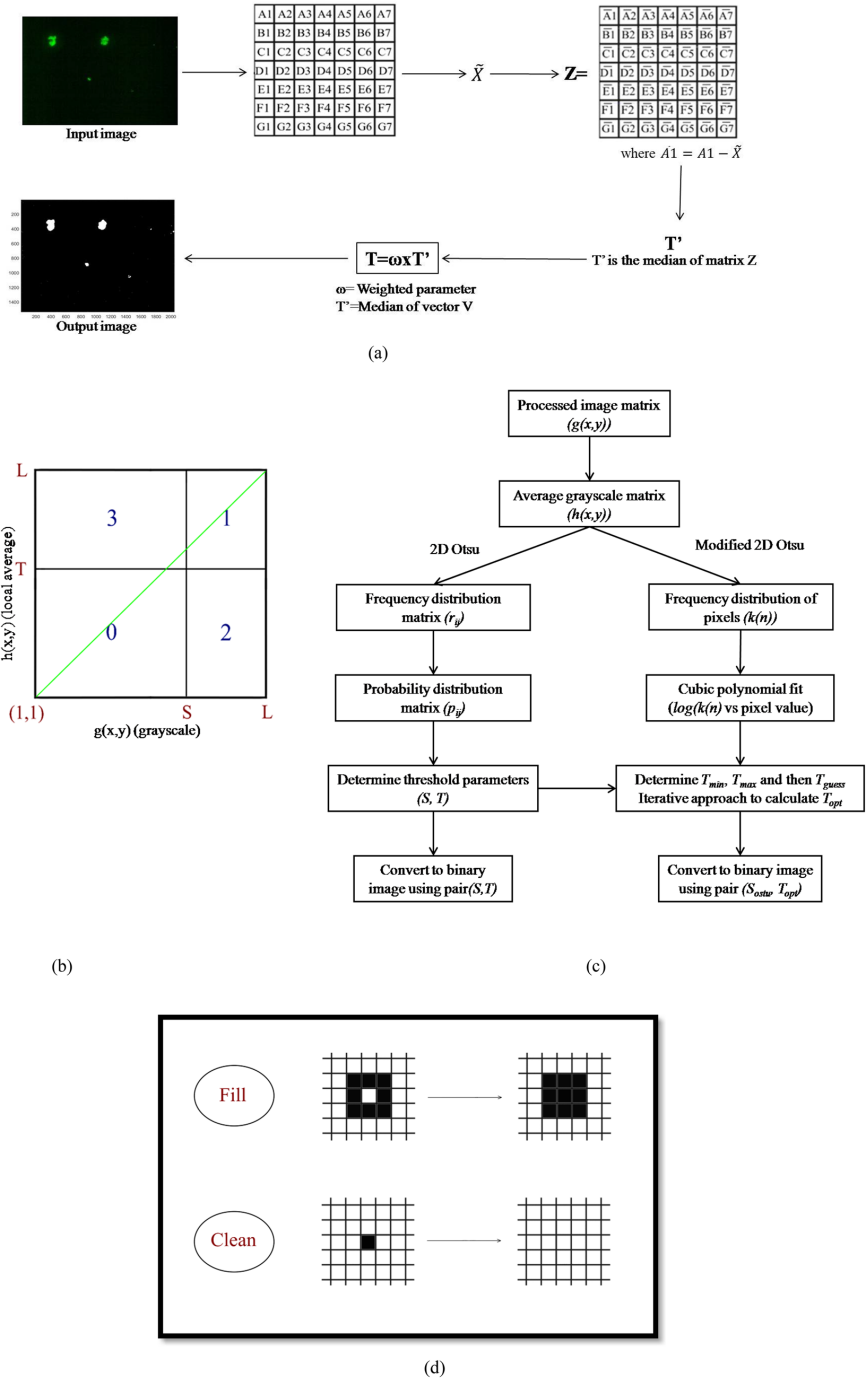
Various steps of median thresholding are shown in (**a**). Further, (**b**) represents the separated classes by the threshold (*S,T)*, (**c**) is the different stages of 2D Otsu and modified 2D Otsu thresholding and (**d**) shows the ‘Fill’ and ‘Clean’ morphological operations.

Final step in image treatment after all the necessary steps of noise reduction is converting it into black and white (binary) image using thresholding method. Level of threshold (*T*) is calculated from different methods available or is user defined. $T$ is compared with the modified image and the pixel values larger than *T* are assigned 1 corresponding to a white pixel. Pixel values less than threshold are marked 0 which corresponds to black pixel as shown in Eq. (5). The different modes of thresholding used are shown in Fig. 3a–c.

5 $$f = \left\{ {\begin{array}{*{20}c} { 0 :g\left( {x,y} \right) < T} \\ { 1 :g\left( {x,y} \right) \ge T.} \\ \end{array} } \right.$$

#### Weighted median thresholding

Median thresholding method requires only one parameter $\left( T \right)$ for obtaining the binary image. Method involves calculation of the median of the complete matrix, the resulting median is subtracted from the image matrix to obtain the deviation with median. Threshold $\left( T \right)$ is equal to the median of the matrix obtained multiplied with the weight. The weight used in the threshold calculation is user defined parameter. The method is explained with the help of Fig. 3a.

#### 1D Otsu thresholding

1D Otsu thresholding calculates the gray level threshold $\left( T \right)$ for binary image conversion. The probability distribution function of the histogram of pixel values is evaluated and then zeroth and first order cumulative moments are calculated using this function. The within class, between class and total variance are evaluated to measure the goodness of the chosen threshold. Optimal threshold $\left( T \right)$ is one for which the between class variance is maximum. The calculated threshold is used to obtain the binary image using Eq. (5) ^19^.

#### 2D Otsu thresholding

2D thresholding approach converts to binary image with the help of two threshold parameters $\left( {S,T} \right)$. In 2D Otsu thresholding method, $S$ and $T$ corresponds to grayscale and average grayscale threshold, respectively. First, the average grayscale matrix $\left( {h\left( {x,y} \right)} \right)$ is calculated using Eq. (6a).

6a $$h\left( {x,y} \right) = \left( {\frac{1}{{n^{2} }}} \right)\mathop \sum \limits_{{i = - \frac{n}{2}}}^{\frac{n}{2}} \mathop \sum \limits_{{j = - \frac{n}{2}}}^{\frac{n}{2}} g\left( {x + i,y + j} \right)n \le N,$$

where, $g\left( {x,y} \right)$ the grayscale/digital image is obtained from the noise removing steps.

Dimension of $g\left( {x,y} \right)$ and $h\left( {x,y} \right)$ is $J$ × $K$. $L$ is the maximum value of the pixel in $g\left( {x,y} \right)$ or $h\left( {x,y} \right)$.

6b $$L = {\text{max}}\left( {g\left( {x,y} \right),h\left( {x,y} \right)} \right).$$

Joint frequency distribution $\left( {r_{ij} } \right)$ is determined for the pair $\left( {i,j} \right)$ for all positions $\left( {x,y} \right)$ where $g\left( {x,y} \right) = i$, $h\left( {x,y} \right) = j, 0 \le r_{ij} \le \left( {JK} \right)$. The dimension of the resulting joint frequency distribution is $L$ × $L$.

From the $r_{ij}$, joint probability distribution $\left( {p_{ij} } \right)$ is calculated using Eq. (7).

7 $$p_{ij} = \frac{{r_{ij} }}{JK}.$$

Joint probability distribution matrix is the basis for calculating within-class, between-class and total variance. The background class ($$C_{0} )$$ and image class $$\left( {C_{1} } \right)$$ are separated by the threshold $$\left( {s,t} \right)$$, the probabilities of class occurrence are given by Eqs. (8) and (9). Figure 3b represents the separated classes by the threshold $$\left( {S,T} \right)$$. The quadrants 0 and 1 in Fig. 3b correspond to background and object classes, respectively, while the quadrants 2 and 3 correspond to pixels near edges and noise^20^. The probability distribution values in quadrants 2 and 3 are negligible because of the use of noise removal and edge enhancing techniques.

8 $$\omega_{0} \left( {s,t} \right) = P_{r} \left( {C_{0} } \right) = \mathop \sum \limits_{i = 1}^{s} \mathop \sum \limits_{j = 1}^{t} p_{ij} ,$$

9 $$\omega_{1} \left( {s,t} \right) = P_{r} \left( {C_{1} } \right) = \mathop \sum \limits_{{i = \left( {s + 1} \right)}}^{L} \mathop \sum \limits_{{j = \left( {t + 1} \right)}}^{L} p_{ij} ,$$

where,$\left( {s,t} \right)$ is the threshold for grayscale and average grayscale.

**Figure 4:**
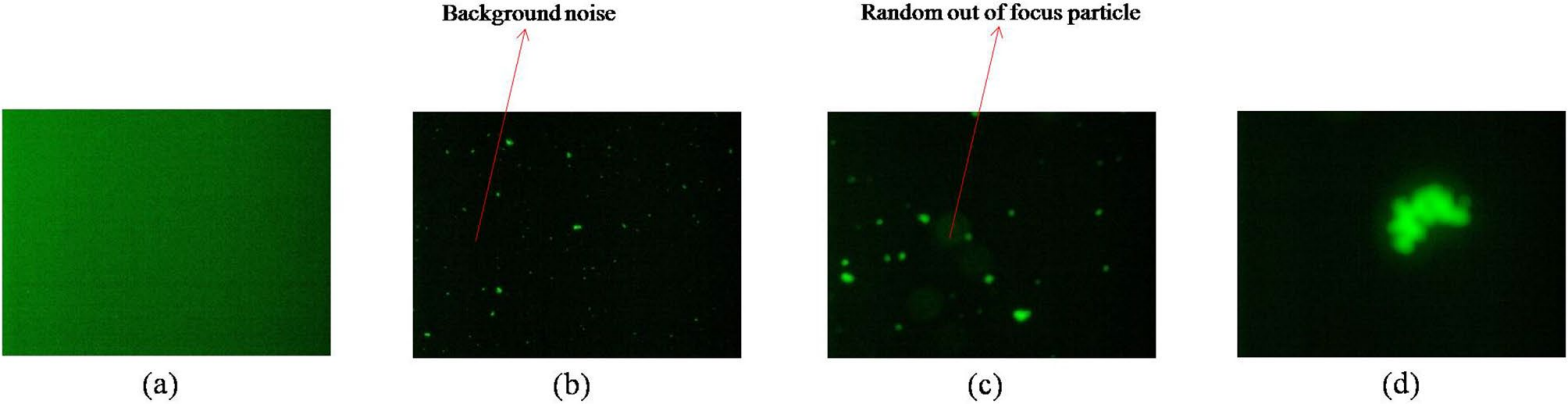
(**a**) is the image of a sample without any mAb (blank). (**b**) and (**c**) show two images of aggregates of various sizes at 4 × and 10 × magnifications and (**d**) is 4 × magnified image showing large sized aggregate.

The mean levels corresponding to each class are defined by Eqs. (10) and (11).

10 $$\mu_{0} = \left( {\mu_{0i} ,\mu_{0j} } \right)^{T} = \left( {\mathop \sum \limits_{i = 1}^{s} iP_{r} \left( {i/C_{0} } \right),\mathop \sum \limits_{j = 1}^{t} jP_{r} \left( {j/C_{0} } \right))} \right)^{T} = \left( {\frac{{\mu_{i} \left( {s,t} \right)}}{{\omega_{0} \left( {s,t} \right)}},\frac{{\mu_{j} \left( {s,t} \right)}}{{\omega_{0} \left( {s,t} \right)}}} \right)^{T} ,$$

where,$$\mu_{i} \left( {s,t} \right) = \sum\nolimits_{i = 1}^{s} {\sum\nolimits_{j = 1}^{t} i } p_{ij} \,and\,\mu_{j} \left( {s,t} \right) = \sum\nolimits_{i = 1}^{s} {\sum\nolimits_{j = 1}^{t} j } p_{ij}$$

11 $$\mu_{1} = \left( {\mu_{1i} ,\mu_{1j} } \right)^{T} = \left( {\mathop \sum \limits_{i = s + 1}^{L} iP_{r} \left( {i/C_{1} } \right),\mathop \sum \limits_{j = t + 1}^{L} jP_{r} \left( {j/C_{1} } \right)} \right)^{T} = \left( {\mathop \sum \limits_{i = s + 1}^{L} \mathop \sum \limits_{j = t + 1}^{L} \frac{{ip_{ij} }}{{\omega_{1} \left( {s,t} \right)}},\mathop \sum \limits_{i = s + 1}^{L} \mathop \sum \limits_{j = t + 1}^{L} \frac{{jp_{ij} }}{{\omega_{1} \left( {s,t} \right)}}} \right)^{T} .$$

The between-class variance $(\sigma_{B} )$ matrix is then calculated using Eq. (12).

12 $$\sigma_{B} = \mathop \sum \limits_{k = 0}^{1} P_{r} \left( {C_{k} } \right)\left[ {\left( {\mu_{k} - \mu_{T} } \right)\left( {\mu_{k} - \mu_{T} } \right)^{T} } \right].$$

Trace of between-class variance $\left( {t_{r} \sigma_{B} } \right)$ is the measure of between-class variance and the threshold $\left( {S,T} \right)$ is one for which the $t_{r} \sigma_{B}$ is maximum as shown by Eqs. (13a) and (13b). Stepwise approach for 2D Otsu thresholding is shown in Fig. 3c^20^.

13a $$t_{r} \sigma_{B} = \frac{{\left[ {\mu_{Ti} \omega_{0} \left( {s,t} \right) - \mu_{i} \left( {s,t} \right)} \right]^{2} + \left[ {\mu_{Tj} \omega_{0} \left( {s,t} \right) - \mu_{j} \left( {s,t} \right)} \right]^{2} }}{{\omega_{0} \left( {s,t} \right)\left[ {1 - \omega_{0} \left( {s,t} \right)} \right]}},$$

13b $$t_{r} \sigma_{B} \left( {S,T} \right) = \max \left\{ {t_{r} \sigma_{B} \left( {s,t} \right)} \right\}.$$

#### Modified 2D Otsu thresholding

Average grayscale threshold parameter $\left( T \right)$ is optimised in this method by extending the 2D otsu algorithm. Optimised grayscale threshold is calculated using the iterative approach. A rough estimate of the total number of particles in the image is required in this method to define convergence criteria. The threshold parameter corresponding to grayscale matrix is the same as obtained from the 2D Otsu method. Frequency distribution, $\left( {k\left( n \right)} \right),{\text{ wherenis the corresponding pixelvalue}})$ vector is first calculated for the average grayscale matrix calculated in the 2D Otsu method (Eq. 6a). Common logarithm of frequency vector $(log\left( {k\left( n \right)} \right)$ is fitted with pixel-value using the cubic polynomial $\left( {P\left( n \right)} \right)$. First derivative $fP\left( n \right)$ is then calculated for the fitted polynomial. Pixel values $\left( {T_{min} ,T_{max} } \right)$ for which the slope of the cubic polynomial is set value are determined. Value of the pixel for which the slope is greater than the set value is termed as minimum threshold $\left( {T_{min} } \right)$ and the value for which the slope is less than the ***set*** value is the maximum threshold $\left( {T_{max} } \right)$. Guess threshold corresponding to average grayscale is chosen such that it lies between the $T_{min}$ and $T_{max}$. Guess threshold $\left( {T_{guess} } \right)$ is defined as the weighted average of $T_{min} , T_{max} and T_{otsu}$ (Eq. 14).

14 $$T_{guess} = w_{1} T_{min} + w_{2} T_{max} + w_{3} T_{otsu} ,$$

where,$w_{1} , w_{2} \,and\, w_{3}$ are weight parameters.

Once the guess threshold is known, calculation of optimum average threshold requires an estimate of the total number of particles in the image. The estimate for the total number of particles $\left( {N_{e} } \right)$ is calculated using the weighted median thresholding method. An iterative approach is used to calculate the optimum value with the help of $T_{guess }$ and $N_{e}$. First the total number of particles $\left( {N_{g} } \right)$ is calculated using the guess threshold. Difference between $N_{e}$ and $N_{g}$ is the error, which is then used to calculate *α* (tuning parameter). The new guess threshold is calculated by subtracting *α* from the old guess threshold. Convergence criteria is defined such that either $N_{g}$ equals $N_{e}$ or $N_{g}$ becomes constant for the subsequent iterative steps. Complete approach of the proposed method is shown in Fig. 3c**.**

Binary image $\left( {f\left( {x,y} \right)} \right)$ formation from the two thresholding parameters $\left( {S,T} \right)$ calculated above for the actual image $\left( {g\left( {x,y} \right)} \right)$ and average grayscale image $\left( {h\left( {x,y} \right)} \right)$ respectively is shown in Eq. (15).

15 $$f = \left\{ {\begin{array}{*{20}c} { 1 :g\left( {x,y} \right) \ge S; h\left( {x,y} \right) \ge T} \\ {0 :for\, all \,other \,cases.} \\ \end{array} } \right.$$

### Morphological operations

Morphological operations are applied to remove any unwanted pixels created due to thresholding. This operation is performed using the in-built morphological operation toolbox in MATLAB. From the available operations, clean, close and fill are used on the modified image. Figure 3d shows the functioning of the applied operations. Size threshold is also applied to remove the bunch of particles smaller than a certain size (considered as noise) based on the type of object. Size threshold is a user defined parameter.

### Labeling particles for size, circularity and average circle diameter calculation

Number of particles in the image is determined by labeling each cluster of pixels with a certain number. Labeling is done with the help of bwlabel function in MATLAB. Number of particles/clusters of pixels in the image is equal to the maximum value of the label. Size/area of clusters is determined by calculating the number of pixels corresponding to each label multiplied by the size of each pixel given by Eq. (16). Circularity of the particles is calculated with the help of perimeter and the area using Eq. (17). Average circle diameter is the equivalent circle diameter of the particle defined by Eq. (18).

16 $$Size\,of\,Particle = \hat{N}lb,$$

17 $$Circularity = 4\pi A/P^{2} ,$$

18 $$\overline{\phi } = \sqrt {4A/\pi } ,$$

where,$\widehat{ N}$ is number of pixels corresponding to a label, $l$ is the length and $b$ is the breadth of one pixel and $A$ is the area of the particle. $P$ is the perimeter of the particle which was directly obtained from MATLAB.

## Results and discussion

### Analytical characterization of aggregates

Visual inspection of the stressed samples showed very high turbidity whereas the unstressed sample and blank (1 × PBS) was not turbid at all (Supplementary Fig. 2a). The high turbidity signified the presence of aggregates of the sub-visible and visible size range. The stressed sample was centrifuged at 5000 rpm for 10 min. The SEC chromatogram of the supernatant did not show the presence of any protein indicating that majority of the tagged mAb was degraded (Supplementary Fig. 2b). The size distribution obtained from DLS showed that the aggregate size distribution was beyond the analysis capability of the instrument (Supplementary Fig. 2c). Fluorescence microscope images indicated presence of various green spots indicating the presence of sub-visible and visible aggregates. These aggregates were introduced in the serum in the ratio of 1:20 (v/v) and incubated at 37 °C. Figure 4 shows four images, namely an image devoid of any sample (blank) (Fig. 4a), images of the serum sample spiked with aggregates of 4 × and 10 × magnification showing numerous aggregates of various sizes (Fig. 4b,c) and an image with a large aggregate obtained at 4 × magnification (Fig. 4d). Apart from the aggregates, all these images are full of random minute dots (noises) that gets detected as aggregates on size distribution analysis using various conventional image processing algorithms. It can also be visualized that each of these figures had varying background illumination. Figure 4c showed some out of focus blurred images that needs to be removed effectively. The images consisting of aggregate specimens were taken for image analysis. Blank samples and images not showing the visible presence of aggregates were not considered.

### Image processing

#### Importing, denoising and background reduction

Florescence images imported in MATLAB using the Image Processing Toolbox in the matrix form had a dimension of 1536 × 2048 × 3. The images obtained at 4 × and 10 × magnification had a scale/pixel ratio of 2.667 and 1.0416, respectively. The contribution of green channel data ($g\left( {x,y} \right)$) was taken for processing. The green channel figures corresponding to Fig. 4b,c are shown in Supplementary Fig. 3a,b. Figure 4b,c are used further as representative images to explain various stages of image processing.

**Figure 5:**
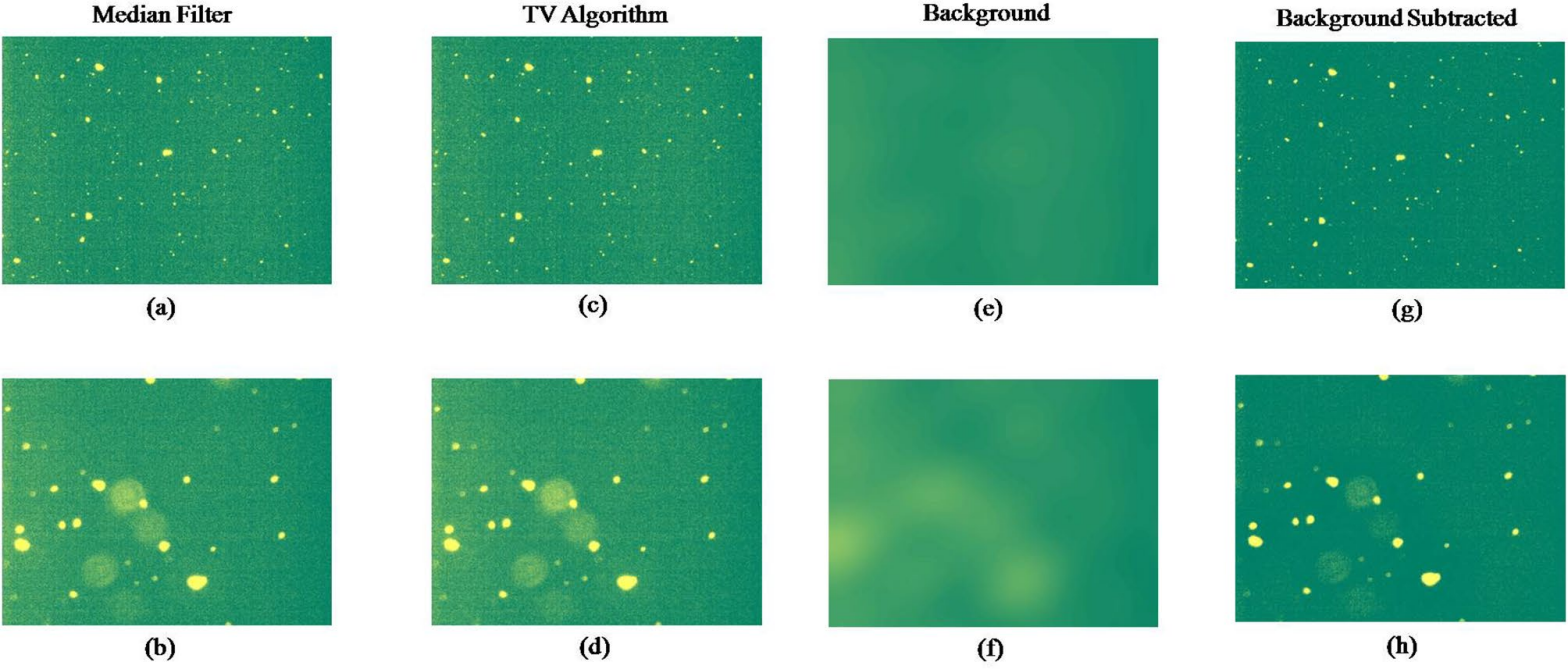
Various images obtained after applying de-noising algorithm on Fig. 4b,c. (**a**) and (**b**) are the median filtered output of Fig. 4b,c. (**c**) and (**d**) are the output after applying TV algorithm, (**e**) and (**f**) are the background and (**g**) and (**h**) are the background subtracted output of Fig. 4b,c.

Filtering is a step in de-noising where a new value is designated for each pixel of the image based on the pixel values of the neighborhood. Median filtering is known to offer advantage over mean filter in that it removes extremely isolated values completely. Median filtering with a 3 × 3 neighborhood was first applied to remove the Gaussian tailing and random noise^16^. Figure 5a shows the image obtained after filtering of Figs. 4b and 5b depicts median filtered output of Fig. 4c. The method is effective in differentiating between the location of particles and background. If the pixel location corresponds to a particle, then most of the neighborhood pixel values are close to the value representing the particle. If the neighborhood of the pixel corresponds to background, then the chosen pixel value is itself background and its value is replaced by the median of the chosen neighborhood.

The aggregates in a mAb sample generally have random shape and size unlike cells. This makes the detection of the edges difficult for aggregates. Since edges of the aggregates are characterized by excessive change in its pixel values as compared to that of background, gradient filters are known to be a useful choice. TV algorithm is a gradient filtering technique which is known to be effective in removing undesired noise in the data along with preserving the information of edges of the particles^21^. Figure 5c,d show the images obtained after processing by reducing the total variance using the TV algorithm on Fig. 5a,b. The algorithm is incorporated in the analysis using gradient descent method^22^. TV(g(x,y)) captures the fluctuations in the image, where higher value represents presence of more noise. The ability to distinguish between the sharp changes due to boundaries of the particles and random fluctuations in the image gives an advantage of using this method for removing noises. The convergence criteria is set such that the relative change in the TV denoised image is less than 0.005 times the Frobenius norm of current guess for g(x,y). On average, 20 steps are enough for convergence using this method.

The illumination noise was addressed by using the Fourier transform for detecting the background of the image. The detected background of the output image (Fig. 5c,d) after being processed by the TV algorithm is shown in Fig. 5e,f. The removed background involved the local signal fluctuations around the target particles which can be observed as light greenish yellow blurry patches. The detected background was then subtracted from the image, which resulted in the images shown in Fig. 5g,h. After the background removal, the image was relatively free from the random background. The normal logarithm frequency distribution plot obtained after background removal is shown in Supplementary Fig. 3c,d. After this step, the image was taken forward for thresholding to convert it into the binary image.

#### Thresholding

Thresholding is done to automatically define the region of interest in the form of a binary image in an unbiased way^23^. The binary image only includes regions that are either the part of the aggregates or background. In case of images without noises, where the target particles are clearly detected, it is possible to apply global thresholding where a lower and upper value of a pixel is chosen to obtain the target image. But in case of noisy images as in our case, mathematical modeling is needed. It is known that in a noisy fluorescence microscope image, a low threshold value retains most of the aggreagate pixels in the binary image and it could enlarge the target particle size and a high threshold value could reduce the number of particles detected in the binary image. This makes the requirement of a suitable algorithm with minimal bias for the required application. Different thresholding methods are discussed and compared here. The two background separated images and the corresponding binary image obtained after weighted median thresholding, 1D Otsu, 2D Otsu, and modified 2D Otsu thresholding are further discussed below. The blank image and its thresholded images along with the size distribution data are shown in Supplementary Fig. 4.

**Figure 6:**
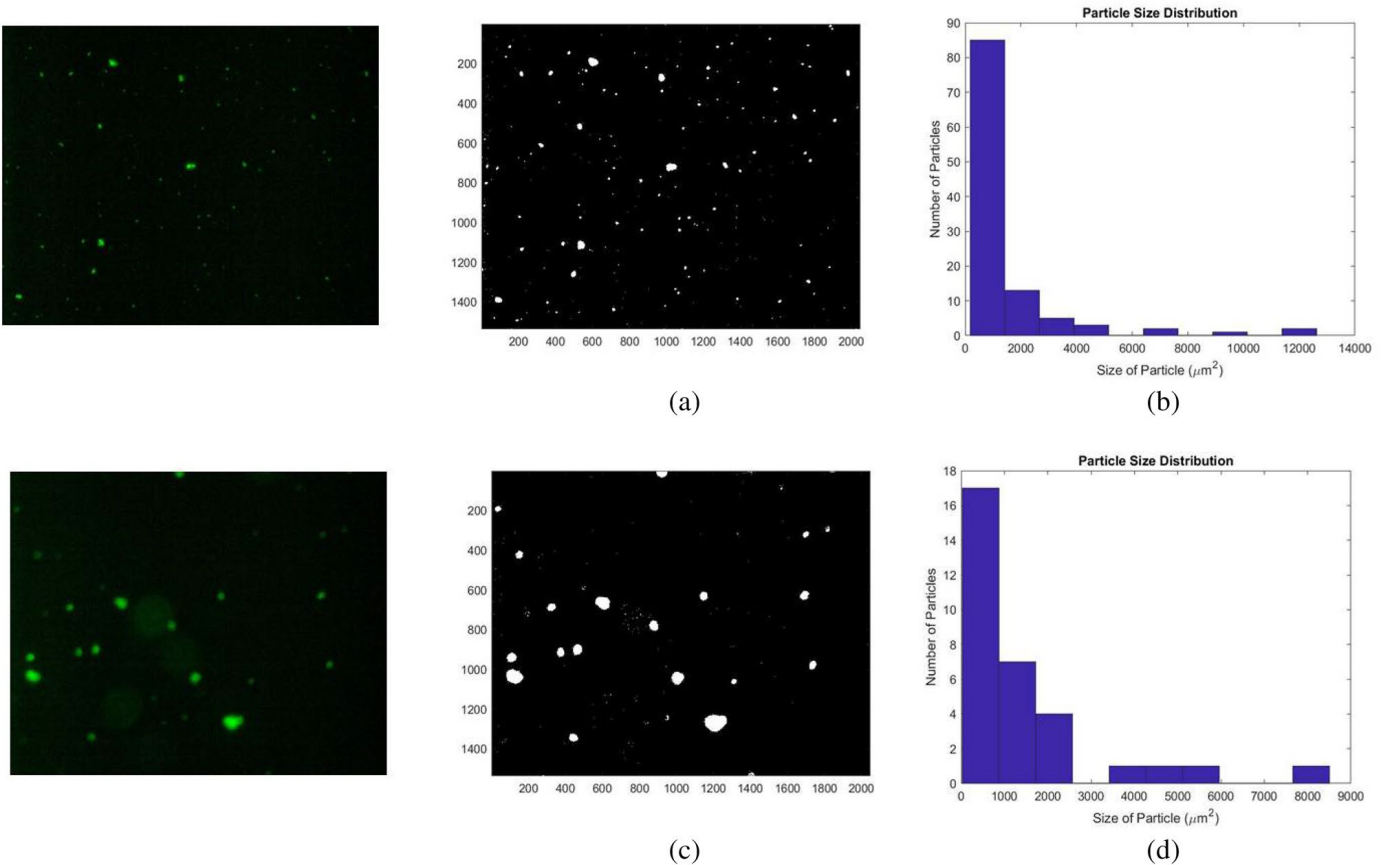
(**a**) and (**b**) are the median thresholded output figure and size distribution graph of Fig. 4b. Similarly (**c**) and (**d**) are the output figure and size distribution graph of Fig. 4c after applying median threshold.

The weight (ω) used to obtain the threshold in the weighted median threshold method was set to be 4.5. Image obtained from the weighted median thresholding is shown in Fig. 6a. The graph showing the particle size distribution as a function of number of particles against size of particles is shown in Fig. 6b. It can be visually observed that most aggregates are correctly identified. The particle size distribution chart further showed the presence of more than 80 particles of area less than 1000 µm^2^ and less than 20 particles for all other sizes. A similar observation can be found for the median thresholded image shown in Fig. 6c. Its size distribution shown in Fig. 6d. The median thresholded blank image and its histogram showed 5 particles being detected. Moreover, median thresholding resulted in blurring and enlarging of target specimens. To avoid the blurring, the utility of Otsu thresholding was explored.

**Figure 7:**
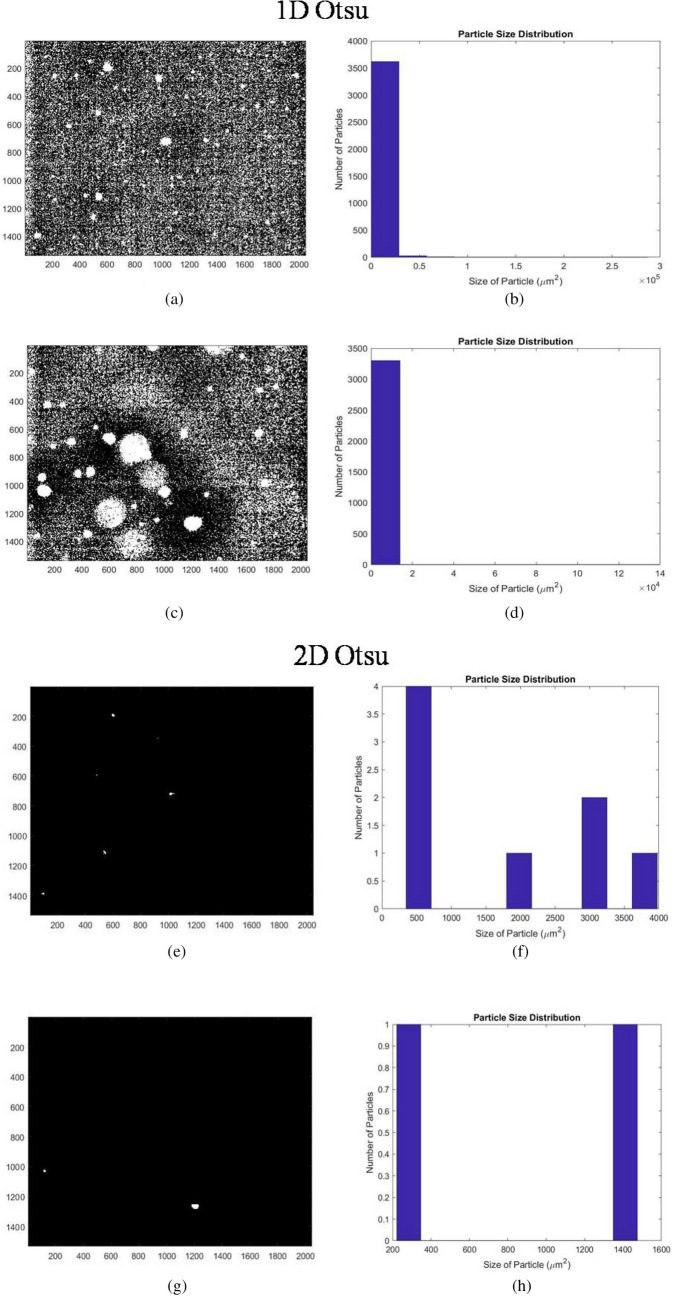
Output of 1D and 2D Otsu thresholding. (**a**) and (**b**) are the 1D Otsu thresholded output figure and size distribution graph of Fig. 4b. (**c**) and (**d**) are the output for Fig. 4c. (**e**) and (**f**) are 2D Otsu thresholded output figure and size distribution graph of Fig. 4b. (**g**) and (**h**) are the 2D Otsu thresholded output figure and size distribution graph of Fig. 4c.

1D Otsu thresholding method is also rigorous in calculating the threshold parameter by comparing the between class variance for each pixel and returns the pixel for which it is maximum. Image and size distribution chart from the 1D Otsu thresholding of Fig. 4b is shown in Fig. 7a,b. It is evident that the image has a lot of grainy appearance which indicates imperfect thresholding due to incorrect detection of particles. The size distribution chart shows the presence of more than 3500 particles of area less than 0.5 µm^2^. The thresholded blank image and its histogram showed numerous grainy disturbances. A similar output after this mode of thresholding for Fig. 4c is shown in Fig. 7c,d. In view of these results, 1D Otsu thresholding was deemed suboptimal for our application.

2D Otsu thresholding can be applied to different types of images. Its applicability is robust for images with background to image ratio near to 1. Figure 7e shows the image obtained after applying 2D Otsu segmentation on Fig. 4b and its size distribution graph is shown in Fig. 7f. The graph shows only 4 detected particles. Majority of the target aggregates got removed resulting in a false negative result. Figure 7g,h, which are the thresholded outputs of Fig. 4c, also resulted in a similar output. Supplementary Fig. 5a shows the output of an image with a large aggregate. Here the aggregate was segmented into two after 2D Otsu thresholding. When the ratio of background to image becomes too small or too large then thresholding using this method is not relevant for the analysis. However, this mode of thresholding prevented blurring as observed in weighted median thresholding and hence can be put into consideration after suitable optimization. Therefore, the parameters obtained from 2D Otsu method were further optimized by modifying the algorithm to achieve better segmentation.

**Figure 8:**
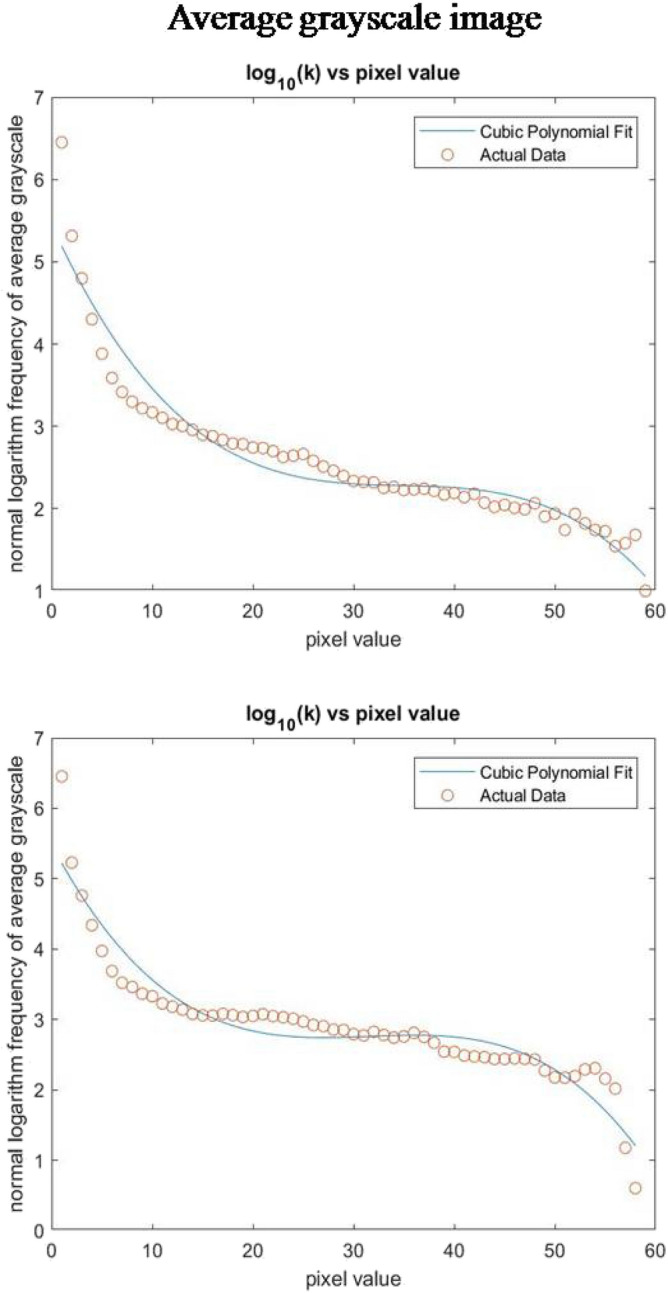
Frequency distribution curves of images in Fig. 4b,c.

The plot of common logarithm of frequency with pixel value for average grayscale matrix $h\left( {x,y} \right)$ for the image (Fig. 4b) is shown in Fig. 8a as actual data. The plot shows that the common logarithm of frequency decreases with increase in pixel values. The decrease in the logarithm of frequency in the plot shows the movement of pixel value from the background to the foreground specimen. The sudden drop in the slope at lower pixel values is a characteristic of the background. This trend is common for all the images as shown in Fig. 8b. This change was captured by fitting a cubic polynomial to the plot as shown in Fig. 8a and then the derivative was calculated. The pair $\left( {T_{min} ,T_{max} } \right)$ were determined when the slope of the fit was − 0.05. The weight parameters were case specific and depended on the type of image needed to be processed. For all of our images or images of fluorescence microscope, weight parameters $w_{1} , w_{2} {\text{and }}w_{3}$ for calculation of $T_{guess}$ were chosen as 0.6, 0.2 and 0.2, respectively. It is known that 2D Otsu method overestimates the value of T, which results in removal of actual particles from the thresholded image. To minimize the overestimation and reduce the number of iterations for optimizing the threshold, more weight was given to the smaller estimate of ${\text{T }}$($T_{min}$). The tuning parameter (α) value was decided by the error as shown in Eq. 19 (Supplementary Fig. 6). The optimized threshold obtained from this method was then used for segmenting the image into binary form using Eq. (15).

**Figure 9:**
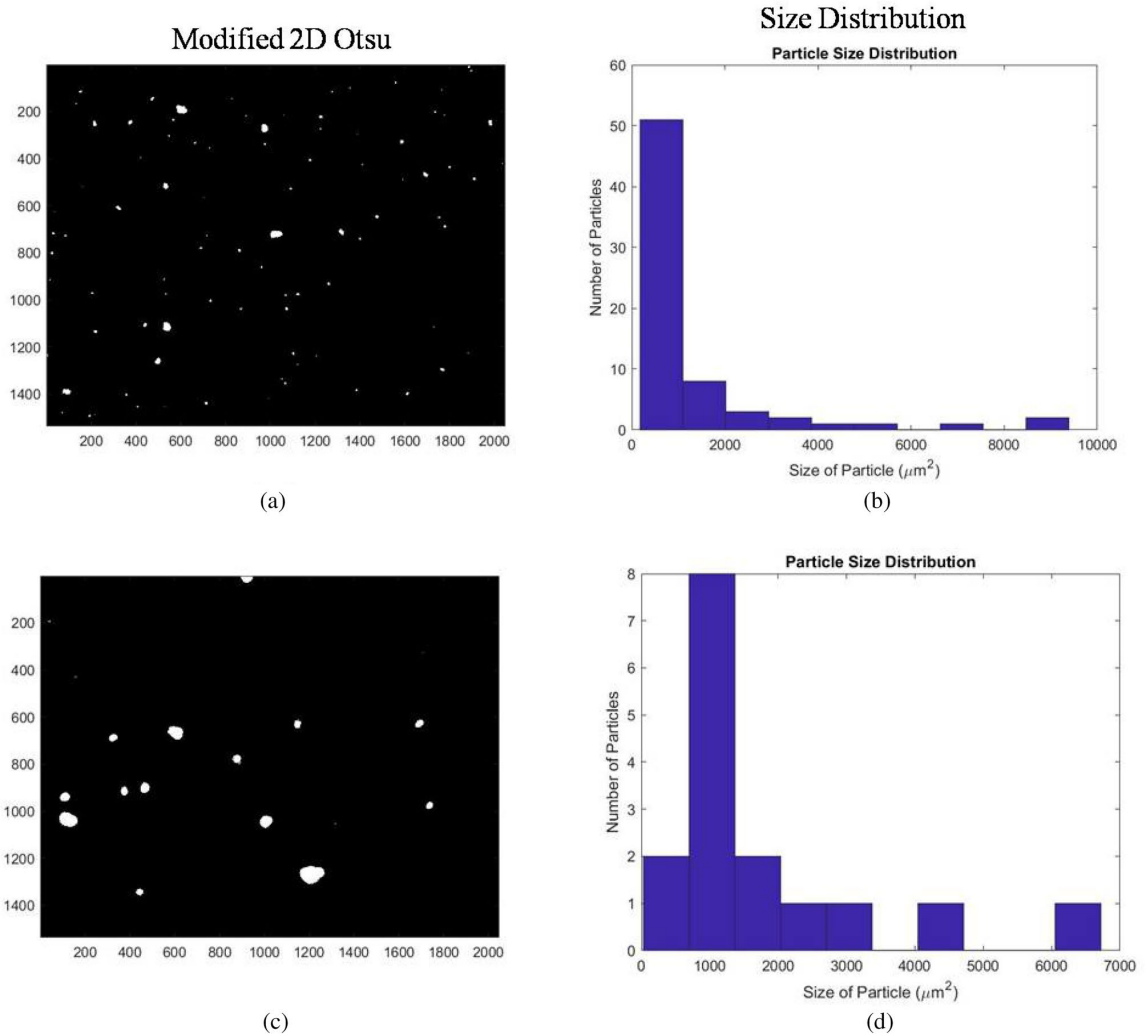
Output of modified 2D Otsu thresholding. (**a**) and (**b**) are the modified 2D Otsu thresholded output figure and size distribution graph for Fig. 4b, whereas (**c**) and (**d**) are the output figure and size distribution for Fig. 4c.

The threshold parameters for this new approach along with other thresholding approaches applied on Fig. 4b,c are mentioned in Table 1. In case of Fig. 4b, the *S* and *T* values were 53 and 9, the $T_{min} , T_{max } and T_{guess}$ were 20.11, 49.48 and 29.56, respectively, and for Fig. 4c, the $S, T,T_{min} , T_{max} andT_{guess}$ values were 57, 9, 17.4, 45.76 and 29.2, respectively. The image obtained after applying modified 2D Otsu thresholding on image in Fig. 4b is shown in Fig. 9a and its size distribution is shown in Fig. 9b. Many of the particles were retained after this thresholding with minimal distortions. However, the number of particles retained were less than weighted median thresholding. The size distribution graph shows that around 50 particles had a size ranging from 0 to 1000 µm^2^ and less than 10 particles observed were of higher size ranges. Similar observations were seen in case of thresholded image shown in Fig. 9c. It is the output for image in Fig. 4c, whose size distribution is represented in Fig. 9d.

**Table 1:** Threshold parameters for Figs. 4b,c.

Our approach		Median thresholding	2D Otsu	1D Otsu	
**Threshold Parameters for Fig. ****4****b**					
*S*	*T*	*S*	*S_otsu*	*T_otsu*	*S*
53	9	26.29	53	38	0.498
T_min	T_max				
20.11	49.48				
T_guess					
29.562					
**Threshold parameters for Fig. ****4****c**					
*S*	*T*	*S*	*S_otsu*	*T_otsu*	*S*
57	9	25.173	57	48	0.498
T_min	T_max				
17.4	45.76				
T_guess					
29.2

It can be summarized that weighted median thresholding retained majority of the aggregates but were slightly blurred. 1D Otsu and 2D Otsu mode of thresholding resulted in over and underestimation of aggregates. Modified 2D Otsu thresholding did result in deletion of some of the aggregates in the image. But many of the aggregates were retained and their sizes were not blurred. Hence, weighted median and modified 2D Otsu mode of thresholding can be considered for further analysis. However, in this study modified 2D Otsu mode of thresholding was taken for morphological operations.

#### Morphological operations and feature extraction

**Figure 10:**
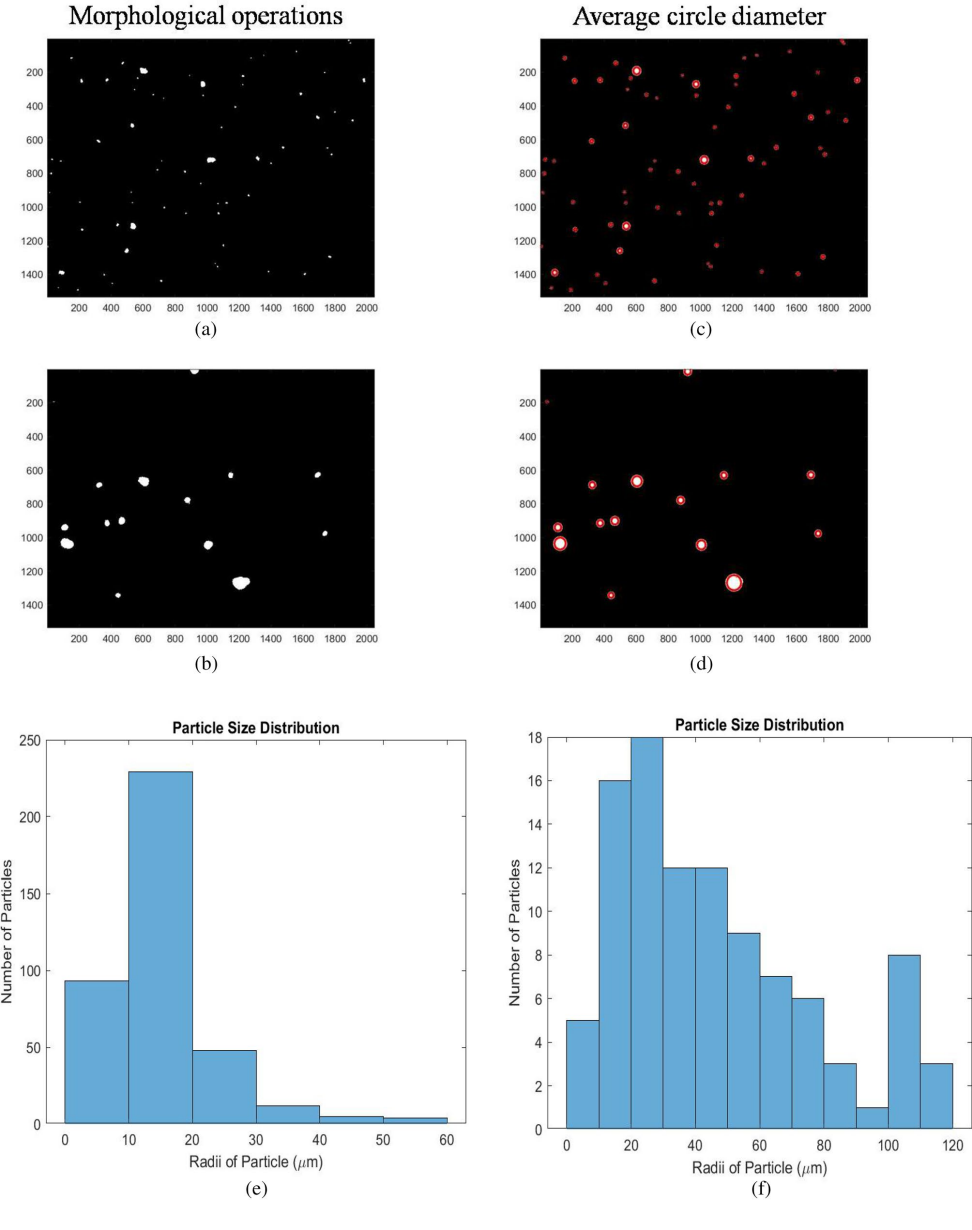
(**a**) and (**b**) are the output after performing morphological operations on Fig. 4b,c. (**c**) and (**d**) shows the images with circles of the corresponding particle’s circularity drawn over the particles. (**e**) and (**f**) shows the **c**omparison of size distribution data of aggregates in serum obtained at 24 and 48 h.

Morphological operations and size-based threshold is applied to the binary image. ‘Clean’ and ‘Fill’ features were used in morphology operations. ‘Clean’ replaces any single isolated pixel of value 1 with 0, while ‘Fill’ replaces all the pixels with value 0 to 1 surrounded with pixels of value 1. A simple size thresholding of 20 pixels was applied to both the images to remove very small sized particles. The 20 pixels corresponded to 142 µm^2^ for 4 × and 21.7 µm^2^ for 10 × or a radius of 6.72 µm for 4 × and 2.62 µm for 10 × magnified images, respectively. The images obtained after performing morphological operations and size thresholding on the output images of modified 2D Otsu thresholding of Fig. 4b,c are shown in Fig. 10a,b. Both the images obtained had morphologically adjusted pixel values with the certain particles removed and added. Now the aggregates to be analyzed were separated from the background and were used for further feature extraction. Number of particles was calculated by using the labeling function in the MATLAB. For each label, the size of the particle was calculated using Eq. (16). The size distribution graphs and images with the circularity of the corresponding particle drawn on the respective particle are shown in Fig. 10c,d. The size (in µm^2^), circularity and radii calculated using the respective scale by pixel ratio for each particle for the images in Fig. 5b is shown in Supplementary Table 1. This raw data was used to construct the size distribution graph. The image processing steps showed that the particle sizes in a fluorescence microscope image can be obtained by removing the characteristic noises. A case study is shown henceforth for the comparison of aggregate sizes in serum over 24 h.

### Case study: analysis of aggregate size using fluorescence microscopy

The size distribution of aggregates spiked into serum was compared at 24 and 48 h. The images were obtained at a magnification of 4x. 20 images of aggregates visualized at 24 h and 4 of them are shown in Supplementary Fig. 7a–d. The images were processed as per the above-mentioned steps and the output after performing morphological operations are shown in Supplementary Fig. 7e–h. The particles and its sizes for all the images were uploaded and combined in MATLAB in the form of a column matrix. These sizes were then arranged in increasing order. The size, perimeter, circularity, and average radii of all the aggregates obtained from the images at 24 h are shown in Supplementary Table 2. Aggregate sizes ranging from 177.82 µm^2^ to 9389.01 µm^2^ were obtained. The average radii of these aggregates varied from 7.52 µm to 54.67 µm. The representative images of the aggregates obtained at 48 h are shown in Supplementary Fig. 8a–d with its corresponding processed images shown in Supplementary Fig. 8e–h. The aggregate size distribution shown in Supplementary Table 3 displayed aggregate sizes ranging from 184.94 µm^2^ (or 7.67 µm radii) to 41,482.36 µm^2^ (114.91 µm radii). These radii were then divided into various ranges of 10 µm from 0 to 10 µm onwards and the number of particles corresponding to each range was plotted. The graphs growing the distribution of average radii at 24 h and 48 h are shown in Fig. 10e,f. It is evident that at 48 h, more aggregates having sizes in the range of 20–30 µm, 40–50 µm to more than 100 µm were present. In case of aggregates obtained at 24 h, majority of aggregates had the size range of 0–10 µm, 10–20 µm and 20–30 µm. The change in the size of aggregates over time was due to either interaction of aggregates among themselves or due to interaction with proteins in serum. Serum consists of albumin (HSA) along with other antigens and antibodies^8,24–26^. The reason behind change in aggregate size on spiking to serum is a matter of further investigation. The comparison of measurable size ranges of aggregates in various microscopic techniques shows that a simple fluorescence microscope can easily analyze aggregates of size ranging from 1 μm (Supplementary Fig. 9).

The 20 images in the case study were randomly captured by the user. However, for more accurate representation of the entire sample, a larger number of images (at least > 150 for magnification of 4×) from multiple slides should be acquired. Further validation is required to optimize the number of images. It should be followed by compilation of outputs from image analysis. Further, fluorescence microscopes capable of automated acquisition of images can be used for capturing such large number of images as these instruments can reduce the manual effort required for capturing the images. An instrument called Cytell Cell Imaging System, capable of acquiring multiple images has been mentioned in the subsequent section.

### Application of other fluorescence-based techniques

The images of samples obtained using CLSM and Cytell showed similar images as seen by fluorescence microscope (Supplementary Fig. 10). The above-mentioned image processing algorithm (with modified 2D Otsu thresholding and a morphological threshold of 7 µm) was applied on a set of 5 images and the obtained average radii was 24.37 µm and 28.4 µm, which was in similar range as compared to the image shown in Fig. 4c (17.08 µm). The accuracy of this data can be further enhanced using automated features of fluorescence based microscopic systems such as Cytell and SDCM (Spinning disk confocal microscopes), that can be helpful in acquiring numerous images per sample. Moreover, using higher magnification such as 20×, 40×, and 60× can be used for more effective visualization and detailed analysis of aggregates. A set of images of the sample containing aggregates were acquired before introducing it into the serum (a representative image in Supplementary Fig. 11), followed by subsequent image analysis. It was found that size distribution of aggregates (in radii) ranged from 1 to 20 µm (Supplementary Fig. 11). Further, the sample containing aggregates were analyzed using Mastersizer 2000 (MS2000, Malvern Instruments, Worcestershire, UK) and it was found the size distribution ranged from 1 to 30 µm (or radii of 0.5–15 µm) (Supplementary Fig. 11c). The results of MS2000 confirms the presence of aggregates in the similar size range as indicated by the image analysis. The use of two different techniques resulted in aggregate sizes of similar range, however the difference in quantitative output can be attributed towards different mechanisms of both^12^. Researchers have demonstrated the fate of aggregates in serum using fSPT, CLSM, AUC with FDS, and FCM^3,6,9,27–29^. These techniques have certain drawbacks. For example, fSPT can measure fluorescent particles only up to 1 μm^6^. FCM requires optimization of instrument settings, use of standard particles and the sample gets diluted in the instrument fluid^3,12^. In case of AUC, the application of centrifugal force might change the nature of aggregates^27^. The current approach provides an easier and faster methodology for size distribution analysis. Further, this methodology does not require any sample dilutions or extensive optimization of instrument settings (Supplementary Table 4).

Apart from analyzing the size distribution of aggregates in serum, the proposed algorithm with fluorescence microscopy can also be used as a tool to analyze visible and sub-visible aggregates. The methodology offers significant advantages over other microscopy-based approaches. Microscopic techniques such as electron and atomic force microscopy can resolve aggregates ranging from 0.1 to 1 nm, whereas various optical and flow imaging microscopes can visualize aggregates with a resolution of 0.5–1 µm^4–6^. Aggregates sizing from 1 µm onwards can be analyzed using a simple optical microscope but it could be inefficient due to the requirement of complex sample preparation steps and the difficulty to differentiate between protein and non-protein particles^12^. In the case of electron microscopy and AFM, the small imaging area limits the acquisition of information about large sized aggregates. Further, complex sample preparation steps, presence of various salts in the sample, extreme expense, optimization of imaging and data acquisition adds to the complexity^4,12^. In case of flow imaging systems such as FlowCAM (Fluid Imaging Technologies, Maine), Sysmex Flow Particle Image Analyzer (FPIA) (Malvern Instruments, Germany), and Micro-Flow Imaging (MFI, Protein simple, Santa Clara, California), the dilution of sample can change the nature of aggregate. The requirement of a certain difference in refractive index between the solvent and the protein could result in inaccurate quantification for certain samples^4,5,12^. Analysis using fluorescence microscopy enables prevention of false detection of dust, air bubbles and non-proteinaceous particles that could plague the analysis^11^. Overall, it can be concluded that the proposed algorithm can successfully denoise the fluorescence microscope images. The proposed methodology can be effectively used as a cheap and complementary technique to traditional approaches.

## Materials and methods

### Chemicals and reagents

High purity high performance liquid chromatography (HPLC) grade chemicals were used throughout the study. All the buffers were prepared in house and were filtered through a 0.22 µm nylon membrane filter (Pall Life Sciences, Port Washington, NY, USA) followed by degassing.

### mAb

An IgG1 mAb having a pI of 8.5 present in 15 mM sodium phosphate, 150 mM NaCl, and 0.1% sodium azide at pH 7.0 was used in the present study. The mAb was donated to us by a major domestic biotech manufacturer. It was stored in − 80 °C until used in experiments.

### Fluorescent labeling of mAb

The mAb used in this study was tagged with FITC. The dye has an emission/excitation peak wavelength of 519/495 nm, giving it a green color. The mAb was labeled with FITC as per the manufacturer’s protocol with minor differences. 1 mg/mL of FITC was prepared in dimethyl sulfoxide (DMSO). The mAb was exchanged to carbonate buffer of pH 8 using 10 kDa Centricon (Pall Corporation, USA) filter followed by addition of dye. The mixture was very gently mixed and incubated at room temperature for 90 min in a falcon tube wrapped with aluminum foil. Then the unbound dye was removed by exchanging the carbonate buffer with a working buffer (1 × PBS) using a 10 kDa Centricon filter.

### Preparation of mAb samples containing aggregates

A 1.5 mL sample containing labelled mAb (1 mg/mL) was subjected to stirring with a magnetic bead in a 5 mL glass vial for 24 h at 4 °C.

### Human serum

Blood samples from 15 healthy individuals were obtained from the local hospital and were pooled together in serum separator tube. Informed written consent was obtained from all participants for drawing their blood. The blood was incubated at room temperature followed by centrifugation at 1000×*g* for 10 min. The supernatant (serum) was separated and transferred into cryovials. The cryovials containing serum was stored at − 80 °C until further required. The experimental protocol was reviewed and approved by the Ethical Committee (Ethics application P-041, IEC, IIT Delhi). All experiments were performed in accordance with relevant guidelines and regulations.

### Analytical SEC

A Thermoscientific Dionex Ultimate 3000 HPLC (Thermo Scientific, Sunnyvale, CA, USA) was used for SEC. The system consisted of a quaternary pump with degasser, auto sampler with cooling unit and a variable wavelength detector (VWD). A Superdex 200 column (GE Healthcare, Pittsburgh, PA, USA) having 30 cm length and 10 mm diameter was used. The system was operated at 25 °C. Mobile phase consisted of a 50 mM phosphate buffer and 300 mM NaCl at pH 6.8. Nylon filter (Pall Corporation) of 0.22 μm cut off was used to filter the mobile phase. Analytes were detected by monitoring UV absorbance at 280 nm. The elution was performed in isocratic mode. The area under the peak was calculated using Chromeleon software (Thermo Scientific, Sunnyvale, CA, USA).

### Visual observation

The stressed samples in the glass vial were visually inspected. The presence of turbidity and visible particles were noted. Vials containing 1 × PBS without and with unstressed monomers were taken as the reference.

### Dynamic light scattering

DLS was performed using Zetasizer Nano ZS 90 (Malvern Instruments), which had a 633-nm He–Ne laser and temperature control. The analysis was performed at 25 °C and 1 mg/mL of sample was used and the scattered intensities were recorded at a scattering angle of 90°.

### Fluorescence microscope

A fluorescence microscope (Olympus IX73) was used to visualize the tagged mAb and aggregates. 20 μL FITC tagged sample was mounted on a clean glass slide followed by covering it with a cover slip. It was then examined under the FITC filter in the microscope at 4 × and 10 × magnification. Various fields of different slides were examined, and the images were captured at different time points.

As an orthogonal tool, samples were also analyzed using Confocal Laser Scanning Microscope (CLSM) and Cytell. Samples containing aggregates were visualized directly under CLSM (Olympus FLUOVIEW FV1200 Confocal Laser Scanning Microscope) at 20 × magnification. Aggregates were further visualized using Cytell Cell Imaging System (GE Healthcare, Buckinghamshire, UK). The Automated Imaging BioApp was used for automatic acquisition of multiple images. Samples containing aggregates in buffer were also analyzed using Mastersizer 2000 (Malvern Instruments, Worcestershire, UK).

## Conclusions

The role of a novel image processing algorithm to analyze fluorescence microscope images was shown to monitor the fate of aggregates of therapeutic IgG samples in serum. Gaussian and Poisson noises along with other random disturbances were effectively removed from fluorescence microscope images using a sequence of de-noising and thresholding techniques. Reducing the total variance along with preserving important information in images was achieved with TV algorithm. A new variant of 2D Otsu thresholding was proposed and found to be highly effective in capturing the changes from background class to image class. Optimization was achieved by an iterative approach using the tuning parameter. The proposed thresholding method is effective in retaining the target particle size. Hence the proposed algorithm was demonstrated as a tool to monitor the effect of aggregates in serum.

## Supplementary Information

Supplementary Information.

## Abbreviations

mAb: Monoclonal antibodies
kDa: Kilodalton
SEC: Size exclusion chromatography
NTA: Nanoparticle tracking analysis
DLS: Dynamic light scattering
FFF: Asymmetrical field-flow fractionation
LO: Light obscuration
AUC: Analytical ultracentrifugation
MFI: Micro flow imaging
PBS: Phosphate buffered saline
HPLC: High performance liquid chromatography
RGB: Red green blue
CLSM: Confocal laser scanning microscopy
FITC: Fluorescein isothiocyanate
3D: 3-Dimensional
1D: 1-Dimensional
2D: 2-Dimensional
DMSO: Dimethyl sulfoxide
NaCl: Sodium chloride
pI: Isoelectric point
SEM: Scanning electron microscopy
TEM: Transmission electron microscopy
AFM: Atomic force microscopy
TV: Total variation
SNR: Signal-by-noise ratio
VWD: Variable wavelength detector
SDCM: Spinning disk confocal microscopes
MS 2000: Mastersizer 2000
FCM: Flow cytometry
