# Supplementary material for: Novel semi-automated fluorescence microscope imaging algorithm for monitoring IgG aggregates in serum
Source: Sci Rep. 2021 May 31;11:11375. doi: 10.1038/s41598-021-90623-7 (PMC8166854; doi:10.1038/s41598-021-90623-7)
Supplement: Supplementary file 1 — Supplementary Information. [file 41598_2021_90623_MOESM1_ESM.docx]

**Supplementary Information**

**Novel semi-automated fluorescence microscope imaging algorithm for monitoring IgG aggregates in serum**

**Authors**

Shravan Sreenivasan^a^, Deepak Sonawat^a^, Shyamapada Mandal^a^, Kedar Khare^b^, Anurag S. Rathore^a*^

**1. List of SI Figures**

SI Figure 1: An example of fluorescence microscope image of aggregates in mAb.

SI Figure 2: SEC chromatogram of sample containing monomer and supernatant of sample stressed by stirring.

SI Figure 3: (a) and (b) are the green channel figures corresponding to Figure 5(b) & (c). Similarly (c) and (d) are the pixel distribution after background removal.

SI Figure 4: Thresholding in a blank image. (a), (b), (c) and (d) shows the various output images after applying various modes of thresholding, whereas (e), (f), (g) and (h) shows the respective size distribution graphs.

SI Figure 5: 2D Otsu and modified 2D Otsu thresholding applied on an image.

SI Figure 6: The set of equations used to find α in the modified 2D Otsu thresholding.

SI Figure 7: Some random images ((a)-(d)) and its corresponding processed images ((e)-(h)) aggregates visualized at 24 hours.

SI Figure 8: Some random representative images ((a)-(d)) and its corresponding processed images ((e)-(h)) of the aggregates obtained at 48 hours in serum.

SI Figure 9: Comparison of measurable size ranges of aggregates in various microscopic techniques.

SI Figure 10: Images of aggregates serum obtained using (a) CLSM and (b) Cytell.

SI Figure 11: An image of aggregates in buffer obtained using fluorescence microscope is shown in (a). The size distribution obtained using image processing is shown in (b) and (c) is the size distribution of sample obtained by MS2000.

SI Figure 1: An example of fluorescence microscope image of aggregate in mAb.


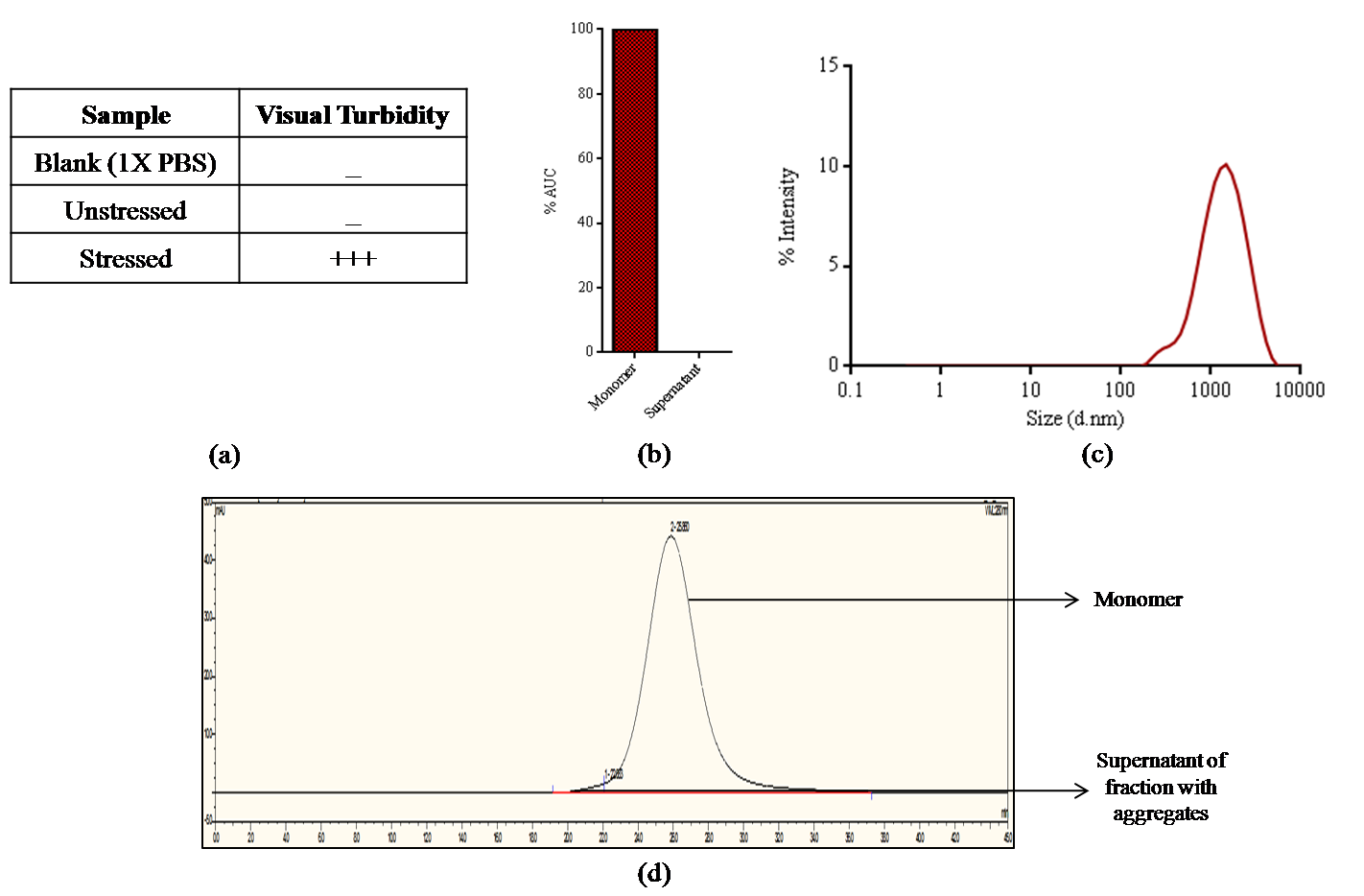


SI Figure 2: (a) is the visually observed turbidity of the samples. (b), (c) and (d) are the relative % area under the curve (AUC) of monomer and stressed sample’s supernatant, DLS size distribution of stressed sample and (d) is the SEC chromatogram of monomer and stressed sample’s supernatant.

SI Figure 3:  (a) and (b) are the green channel figures corresponding to Figure 5(b) & (c). Similarly (c) and (d) are the pixel distribution after background removal.

SI Figure 4: Thresholding in a blank image. (a), (b), (c) and (d) shows the various output images after applying various modes of thresholding, whereas (e), (f), (g) and (h) shows the respective size distribution graphs.

SI Figure 5: 2D Otsu and modified 2D Otsu thresholding applied on an image.


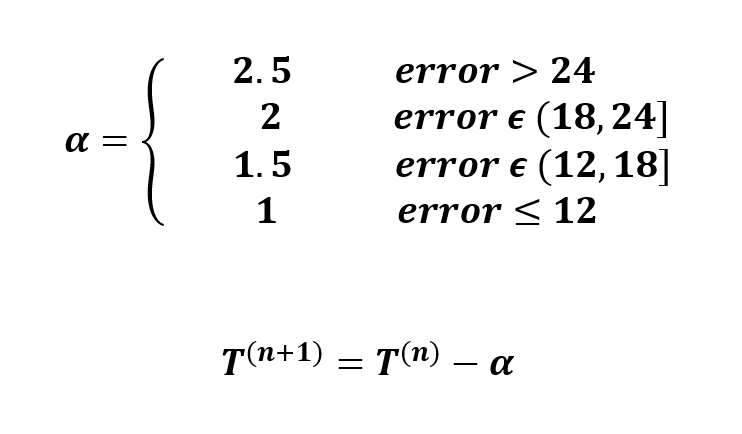


SI Figure 6: The set of equations used to find α in the modified 2D Otsu thresholding.

SI Figure 7: Some random images ((a)-(d)) and its corresponding processed images ((e)-(h)) of aggregates visualized at 24 hours.

SI Figure 8: Some random representative images ((a)-(d)) and its corresponding processed images ((e)-(h)) of the aggregates obtained at 48 hours in serum.


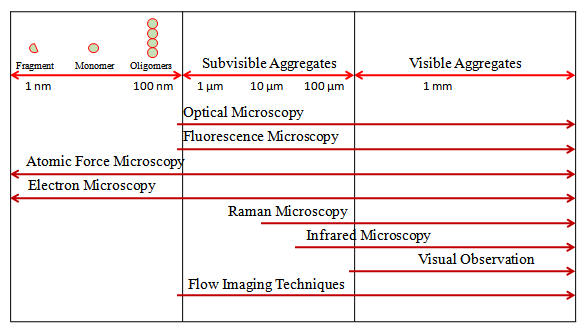


SI Figure 9: Comparison of measurable size ranges of aggregates in various microscopic techniques.


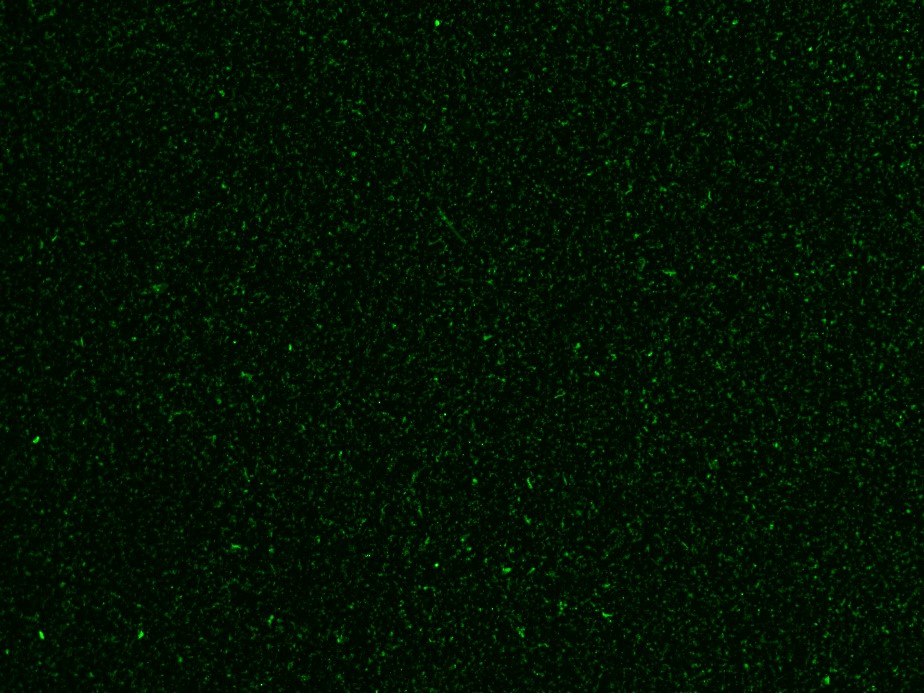

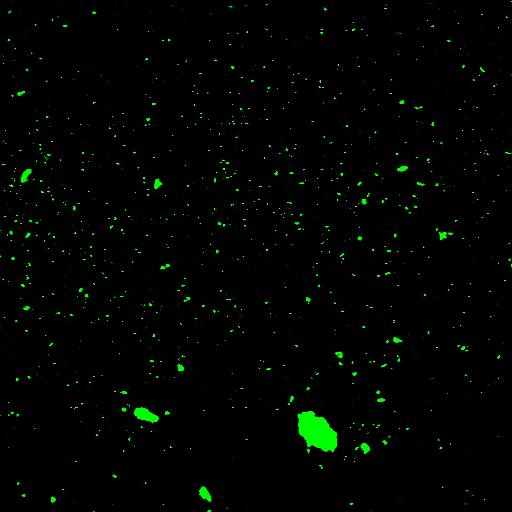


(a)

(b)

SI Figure 10: Images of aggregates serum obtained using (a) CLSM and (b) Cytell.


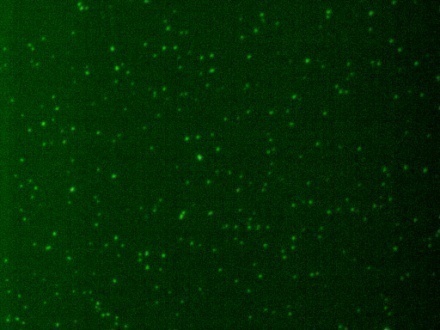


(a)


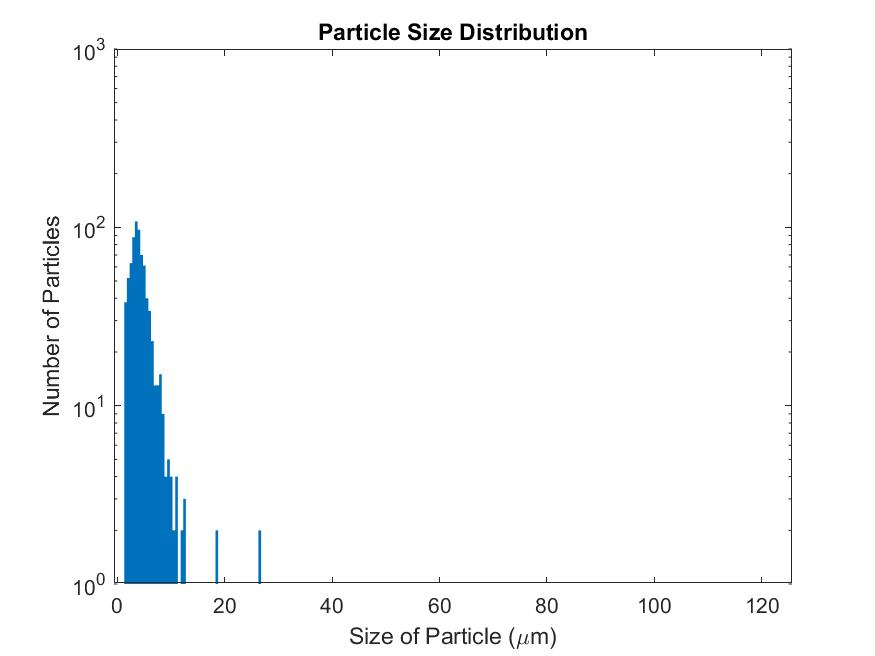


(b)


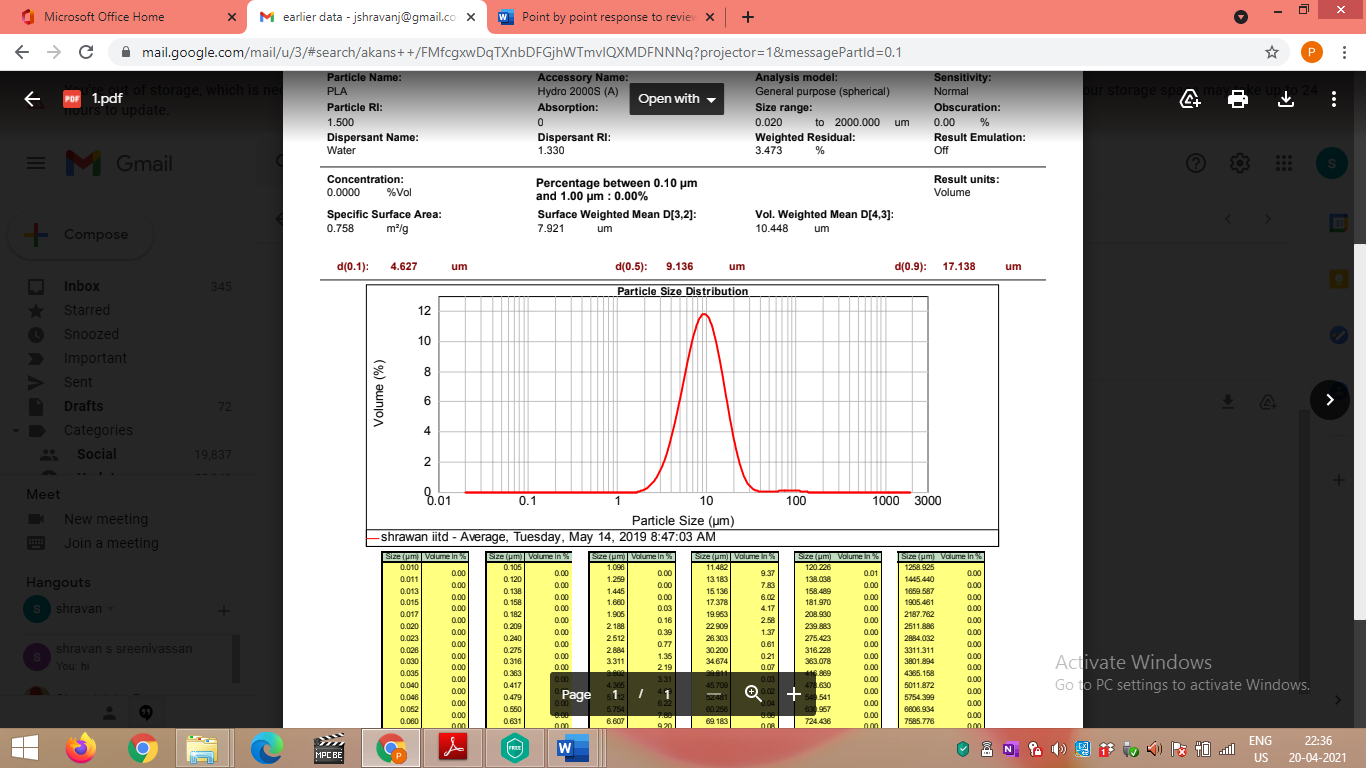


(c)

SI Figure 11: An image of aggregates in buffer obtained using fluorescence microscope is shown in (a). The size distribution obtained using image processing is shown in (b) and (c) is the size distribution of sample obtained by MS2000.

**2. List of SI Tables**

SI Table 1: The area, perimeter, circularity and average radii of each of the aggregates detected in Figure 5(b).

SI Table 2: The size, perimeter, circularity and average radii of all the aggregates in the images obtained at 24 hours.

SI Table 3: The area, perimeter, circularity, and average radii of all the aggregates in the in the images obtained at 48 hours.

SI Table 4: Various techniques and the drawbacks associated with them for analyzing aggregates in IgG sample in serum.

SI Table 1: The area, perimeter, circularity, and average radii of each of the aggregates detected in Figure 5(b).

| **Size (µm^2^)** | **Perimeter (µm)** | **Circularity** | **Average Radii (µm)** |
| --- | --- | --- | --- |
| 426.77 | 77.57 | 0.89 | 11.66 |
| 184.94 | 41.22 | 1.37 | 7.67 |
| 611.71 | 86.06 | 1.04 | 13.95 |
| 540.58 | 77.2 | 1.14 | 13.12 |
| 241.84 | 49.72 | 1.23 | 8.77 |
| 4680.28 | 272.91 | 0.79 | 38.6 |
| 362.76 | 62.93 | 1.15 | 10.75 |
| 668.61 | 92.22 | 0.99 | 14.59 |
| 512.13 | 77.64 | 1.07 | 12.77 |
| 490.79 | 74.62 | 1.11 | 12.5 |
| 1856.46 | 156.07 | 0.96 | 24.31 |
| 1194.97 | 120.12 | 1.04 | 19.5 |
| 1728.43 | 163.57 | 0.81 | 23.46 |
| 576.14 | 83.66 | 1.03 | 13.54 |
| 1835.13 | 152.88 | 0.99 | 24.17 |
| 348.53 | 58.94 | 1.26 | 10.53 |
| 1323 | 129.12 | 1 | 20.52 |
| 1081.16 | 116.22 | 1.01 | 18.55 |
| 3321.72 | 217.9 | 0.88 | 32.52 |
| 3058.54 | 198.69 | 0.97 | 31.2 |
| 7361.84 | 320.33 | 0.9 | 48.41 |
| 263.18 | 49.96 | 1.33 | 9.15 |
| 277.4 | 53.47 | 1.22 | 9.4 |
| 184.94 | 42.94 | 1.26 | 7.67 |
| 554.81 | 75.92 | 1.21 | 13.29 |
| 9389.01 | 387.01 | 0.79 | 54.67 |
| 490.79 | 73.96 | 1.13 | 12.5 |
| 469.45 | 72.65 | 1.12 | 12.22 |
| 924.68 | 102.8 | 1.1 | 17.16 |
| 177.82 | 41.47 | 1.3 | 7.52 |
| 213.39 | 46.45 | 1.24 | 8.24 |
| 633.05 | 83.24 | 1.15 | 14.2 |
| 839.32 | 99.36 | 1.07 | 16.35 |
| 419.66 | 66.46 | 1.19 | 11.56 |
| 184.94 | 44.25 | 1.19 | 7.67 |
| 391.21 | 63.61 | 1.21 | 11.16 |
| 5604.96 | 277.38 | 0.92 | 42.24 |
| 561.92 | 80.64 | 1.09 | 13.37 |
| 9040.48 | 385.73 | 0.76 | 53.64 |
| 220.5 | 46.69 | 1.27 | 8.38 |
| 405.43 | 65.4 | 1.19 | 11.36 |
| 426.77 | 66.46 | 1.21 | 11.66 |
| 817.98 | 97.14 | 1.09 | 16.14 |
| 441 | 68.22 | 1.19 | 11.85 |
| 675.72 | 93.63 | 0.97 | 14.67 |
| 867.77 | 103.91 | 1.01 | 16.62 |
| 668.61 | 85.38 | 1.15 | 14.59 |
| 1152.29 | 117.36 | 1.05 | 19.15 |
| 256.06 | 53.71 | 1.12 | 9.03 |
| 689.95 | 89.46 | 1.08 | 14.82 |
| 234.73 | 48.48 | 1.25 | 8.64 |
| 2610.43 | 192.91 | 0.88 | 28.83 |
| 184.94 | 40.98 | 1.38 | 7.67 |
| 384.1 | 61.71 | 1.27 | 11.06 |
| 455.22 | 68.66 | 1.21 | 12.04 |
| 1045.59 | 118.95 | 0.93 | 18.24 |
| 312.97 | 54.7 | 1.31 | 9.98 |
| 1394.13 | 158.85 | 0.69 | 21.07 |
| 825.1 | 110.9 | 0.84 | 16.21 |
| 2155.21 | 174.84 | 0.89 | 26.19 |
| 362.76 | 60.24 | 1.26 | 10.75 |
| 519.24 | 81.15 | 0.99 | 12.86 |
| 1387.01 | 133.55 | 0.98 | 21.01 |
| 711.29 | 91.69 | 1.06 | 15.05 |
| 490.79 | 75.99 | 1.07 | 12.5 |
| 448.11 | 70.21 | 1.14 | 11.94 |
| 291.63 | 56.49 | 1.15 | 9.63 |
| 839.32 | 97.64 | 1.11 | 16.35 |
| 2325.91 | 179.96 | 0.9 | 27.21 |

SI Table 2: The size, perimeter, circularity and average radii of all the aggregates in the images obtained at 24 hours.

| **Size (µm^2^)** | **Perimeter (µm)** | **Circularity** | **Average Radii (µm)** |
| --- | --- | --- | --- |
| 177.82 | 40.74 | 1.35 | 7.52 |
| 177.82 | 41.47 | 1.3 | 7.52 |
| 177.82 | 41.71 | 1.28 | 7.52 |
| 177.82 | 39.99 | 1.4 | 7.52 |
| 177.82 | 40.47 | 1.36 | 7.52 |
| 177.82 | 40.67 | 1.35 | 7.52 |
| 177.82 | 40.74 | 1.35 | 7.52 |
| 177.82 | 42.22 | 1.25 | 7.52 |
| 177.82 | 40.47 | 1.36 | 7.52 |
| 177.82 | 43.19 | 1.2 | 7.52 |
| 177.82 | 39.44 | 1.44 | 7.52 |
| 184.94 | 41.22 | 1.37 | 7.67 |
| 184.94 | 42.94 | 1.26 | 7.67 |
| 184.94 | 44.25 | 1.19 | 7.67 |
| 184.94 | 40.98 | 1.38 | 7.67 |
| 184.94 | 47.25 | 1.04 | 7.67 |
| 184.94 | 43.43 | 1.23 | 7.67 |
| 192.05 | 42.7 | 1.32 | 7.82 |
| 192.05 | 41.47 | 1.4 | 7.82 |
| 192.05 | 41.95 | 1.37 | 7.82 |
| 192.05 | 46.45 | 1.12 | 7.82 |
| 192.05 | 43.19 | 1.29 | 7.82 |
| 199.16 | 42.94 | 1.36 | 7.96 |
| 199.16 | 42.22 | 1.4 | 7.96 |
| 199.16 | 44.97 | 1.24 | 7.96 |
| 206.27 | 45.97 | 1.23 | 8.1 |
| 206.27 | 44.97 | 1.28 | 8.1 |
| 206.27 | 45.97 | 1.23 | 8.1 |
| 206.27 | 45.22 | 1.27 | 8.1 |
| 206.27 | 45.72 | 1.24 | 8.1 |
| 206.27 | 46.45 | 1.2 | 8.1 |
| 213.39 | 44.97 | 1.33 | 8.24 |
| 213.39 | 46.45 | 1.24 | 8.24 |
| 213.39 | 46.52 | 1.24 | 8.24 |
| 213.39 | 49.96 | 1.07 | 8.24 |
| 213.39 | 50.13 | 1.07 | 8.24 |
| 220.5 | 46.69 | 1.27 | 8.38 |
| 220.5 | 44.66 | 1.39 | 8.38 |
| 227.61 | 49.47 | 1.17 | 8.51 |
| 234.73 | 48.41 | 1.26 | 8.64 |
| 234.73 | 48.48 | 1.25 | 8.64 |
| 241.84 | 48.41 | 1.3 | 8.77 |
| 241.84 | 49.72 | 1.23 | 8.77 |
| 241.84 | 47.93 | 1.32 | 8.77 |
| 241.84 | 49.47 | 1.24 | 8.77 |
| 248.95 | 49.23 | 1.29 | 8.9 |
| 248.95 | 52.23 | 1.15 | 8.9 |
| 248.95 | 52.43 | 1.14 | 8.9 |
| 256.06 | 52.23 | 1.18 | 9.03 |
| 256.06 | 53.71 | 1.12 | 9.03 |
| 256.06 | 49.72 | 1.3 | 9.03 |
| 256.06 | 48.97 | 1.34 | 9.03 |
| 256.06 | 49.96 | 1.29 | 9.03 |
| 263.18 | 51.92 | 1.23 | 9.15 |
| 263.18 | 49.96 | 1.33 | 9.15 |
| 263.18 | 49.96 | 1.33 | 9.15 |
| 263.18 | 51.68 | 1.24 | 9.15 |
| 263.18 | 55.98 | 1.06 | 9.15 |
| 263.18 | 53.88 | 1.14 | 9.15 |
| 263.18 | 56.73 | 1.03 | 9.15 |
| 270.29 | 53.73 | 1.18 | 9.28 |
| 270.29 | 53.95 | 1.17 | 9.28 |
| 270.29 | 54.7 | 1.14 | 9.28 |
| 270.29 | 55.19 | 1.12 | 9.28 |
| 270.29 | 53.16 | 1.2 | 9.28 |
| 277.4 | 53.71 | 1.21 | 9.4 |
| 277.4 | 53.47 | 1.22 | 9.4 |
| 277.4 | 52.67 | 1.26 | 9.4 |
| 277.4 | 55.43 | 1.13 | 9.4 |
| 277.4 | 53.95 | 1.2 | 9.4 |
| 277.4 | 59.18 | 1 | 9.4 |
| 277.4 | 54.94 | 1.15 | 9.4 |
| 277.4 | 55.98 | 1.11 | 9.4 |
| 284.52 | 53.71 | 1.24 | 9.52 |
| 284.52 | 51.44 | 1.35 | 9.52 |
| 284.52 | 55.25 | 1.17 | 9.52 |
| 284.52 | 56.24 | 1.13 | 9.52 |
| 291.63 | 53.71 | 1.27 | 9.63 |
| 291.63 | 53.22 | 1.29 | 9.63 |
| 291.63 | 56.49 | 1.15 | 9.63 |
| 291.63 | 52.91 | 1.31 | 9.63 |
| 291.63 | 52.91 | 1.31 | 9.63 |
| 298.74 | 55.91 | 1.2 | 9.75 |
| 298.74 | 62.88 | 0.95 | 9.75 |
| 298.74 | 57.46 | 1.14 | 9.75 |
| 298.74 | 56.73 | 1.17 | 9.75 |
| 305.85 | 57.66 | 1.16 | 9.87 |
| 312.97 | 54.7 | 1.31 | 9.98 |
| 312.97 | 54.7 | 1.31 | 9.98 |
| 312.97 | 56.91 | 1.21 | 9.98 |
| 312.97 | 63.99 | 0.96 | 9.98 |
| 312.97 | 59.18 | 1.12 | 9.98 |
| 312.97 | 63.19 | 0.98 | 9.98 |
| 320.08 | 56.97 | 1.24 | 10.09 |
| 320.08 | 58.25 | 1.19 | 10.09 |
| 320.08 | 60.1 | 1.11 | 10.09 |
| 320.08 | 60.41 | 1.1 | 10.09 |
| 320.08 | 58.94 | 1.16 | 10.09 |
| 320.08 | 59.18 | 1.15 | 10.09 |
| 320.08 | 65.71 | 0.93 | 10.09 |
| 320.08 | 59.93 | 1.12 | 10.09 |
| 327.19 | 70.69 | 0.82 | 10.21 |
| 334.31 | 57.94 | 1.25 | 10.32 |
| 334.31 | 56.66 | 1.31 | 10.32 |
| 334.31 | 71.44 | 0.82 | 10.32 |
| 341.42 | 63.5 | 1.06 | 10.42 |
| 341.42 | 62.71 | 1.09 | 10.42 |
| 341.42 | 62.2 | 1.11 | 10.42 |
| 341.42 | 63.24 | 1.07 | 10.42 |
| 348.53 | 58.94 | 1.26 | 10.53 |
| 348.53 | 60.72 | 1.19 | 10.53 |
| 348.53 | 72.65 | 0.83 | 10.53 |
| 355.64 | 67.43 | 0.98 | 10.64 |
| 355.64 | 60.96 | 1.2 | 10.64 |
| 355.64 | 58.63 | 1.3 | 10.64 |
| 362.76 | 62.93 | 1.15 | 10.75 |
| 362.76 | 60.24 | 1.26 | 10.75 |
| 369.87 | 60.41 | 1.27 | 10.85 |
| 369.87 | 65.95 | 1.07 | 10.85 |
| 369.87 | 64.41 | 1.12 | 10.85 |
| 369.87 | 69.21 | 0.97 | 10.85 |
| 369.87 | 60.72 | 1.26 | 10.85 |
| 369.87 | 63.99 | 1.14 | 10.85 |
| 369.87 | 65.95 | 1.07 | 10.85 |
| 369.87 | 64.85 | 1.11 | 10.85 |
| 369.87 | 66.7 | 1.04 | 10.85 |
| 376.98 | 62.95 | 1.2 | 10.95 |
| 384.1 | 63.68 | 1.19 | 11.06 |
| 384.1 | 61.71 | 1.27 | 11.06 |
| 384.1 | 67.67 | 1.05 | 11.06 |
| 384.1 | 68.22 | 1.04 | 11.06 |
| 391.21 | 63.97 | 1.2 | 11.16 |
| 391.21 | 63.61 | 1.21 | 11.16 |
| 391.21 | 67.96 | 1.06 | 11.16 |
| 391.21 | 63.68 | 1.21 | 11.16 |
| 391.21 | 69.9 | 1.01 | 11.16 |
| 398.32 | 63.68 | 1.23 | 11.26 |
| 398.32 | 65.4 | 1.17 | 11.26 |
| 398.32 | 63.68 | 1.23 | 11.26 |
| 398.32 | 65.46 | 1.17 | 11.26 |
| 405.43 | 64.16 | 1.24 | 11.36 |
| 405.43 | 65.4 | 1.19 | 11.36 |
| 405.43 | 65.95 | 1.17 | 11.36 |
| 405.43 | 72.96 | 0.96 | 11.36 |
| 412.55 | 67.43 | 1.14 | 11.46 |
| 419.66 | 65.15 | 1.24 | 11.56 |
| 419.66 | 66.46 | 1.19 | 11.56 |
| 419.66 | 66.43 | 1.19 | 11.56 |
| 419.66 | 68.73 | 1.12 | 11.56 |
| 426.77 | 67.36 | 1.18 | 11.66 |
| 426.77 | 77.57 | 0.89 | 11.66 |
| 426.77 | 66.46 | 1.21 | 11.66 |
| 433.89 | 76.91 | 0.92 | 11.75 |
| 441 | 68.22 | 1.19 | 11.85 |
| 441 | 80.46 | 0.86 | 11.85 |
| 441 | 73.4 | 1.03 | 11.85 |
| 448.11 | 70.21 | 1.14 | 11.94 |
| 448.11 | 68.71 | 1.19 | 11.94 |
| 448.11 | 71.97 | 1.09 | 11.94 |
| 455.22 | 68.66 | 1.21 | 12.04 |
| 462.34 | 69.15 | 1.22 | 12.13 |
| 469.45 | 70.69 | 1.18 | 12.22 |
| 469.45 | 72.65 | 1.12 | 12.22 |
| 476.56 | 79.36 | 0.95 | 12.32 |
| 483.68 | 74.44 | 1.1 | 12.41 |
| 483.68 | 71.66 | 1.18 | 12.41 |
| 490.79 | 76.16 | 1.06 | 12.5 |
| 490.79 | 71.66 | 1.2 | 12.5 |
| 490.79 | 74.62 | 1.11 | 12.5 |
| 490.79 | 73.96 | 1.13 | 12.5 |
| 490.79 | 75.99 | 1.07 | 12.5 |
| 497.9 | 76.69 | 1.06 | 12.59 |
| 497.9 | 86.37 | 0.84 | 12.59 |
| 497.9 | 74.44 | 1.13 | 12.59 |
| 505.02 | 73.89 | 1.16 | 12.68 |
| 512.13 | 80.4 | 1 | 12.77 |
| 512.13 | 77.64 | 1.07 | 12.77 |
| 512.13 | 81.94 | 0.96 | 12.77 |
| 519.24 | 81.15 | 0.99 | 12.86 |
| 526.35 | 73.14 | 1.24 | 12.94 |
| 526.35 | 79.12 | 1.06 | 12.94 |
| 526.35 | 74.13 | 1.2 | 12.94 |
| 526.35 | 79.93 | 1.04 | 12.94 |
| 533.47 | 79.36 | 1.06 | 13.03 |
| 533.47 | 77.4 | 1.12 | 13.03 |
| 540.58 | 77.2 | 1.14 | 13.12 |
| 540.58 | 78.06 | 1.11 | 13.12 |
| 540.58 | 77.95 | 1.12 | 13.12 |
| 540.58 | 78.46 | 1.1 | 13.12 |
| 540.58 | 77.95 | 1.12 | 13.12 |
| 540.58 | 77.22 | 1.14 | 13.12 |
| 547.69 | 75.92 | 1.19 | 13.2 |
| 547.69 | 78.19 | 1.13 | 13.2 |
| 554.81 | 75.92 | 1.21 | 13.29 |
| 554.81 | 89.97 | 0.86 | 13.29 |
| 554.81 | 78.37 | 1.14 | 13.29 |
| 554.81 | 76.4 | 1.19 | 13.29 |
| 554.81 | 79.18 | 1.11 | 13.29 |
| 554.81 | 78.94 | 1.12 | 13.29 |
| 561.92 | 80.64 | 1.09 | 13.37 |
| 561.92 | 79.6 | 1.11 | 13.37 |
| 569.03 | 80.9 | 1.09 | 13.46 |
| 576.14 | 83.66 | 1.03 | 13.54 |
| 590.37 | 82.93 | 1.08 | 13.71 |
| 597.48 | 84.9 | 1.04 | 13.79 |
| 604.6 | 93.21 | 0.87 | 13.87 |
| 604.6 | 82.14 | 1.13 | 13.87 |
| 611.71 | 86.06 | 1.04 | 13.95 |
| 611.71 | 89.2 | 0.97 | 13.95 |
| 625.93 | 86.55 | 1.05 | 14.12 |
| 625.93 | 84.17 | 1.11 | 14.12 |
| 625.93 | 82.62 | 1.15 | 14.12 |
| 625.93 | 84.46 | 1.1 | 14.12 |
| 633.05 | 83.24 | 1.15 | 14.2 |
| 640.16 | 85.89 | 1.09 | 14.27 |
| 640.16 | 85.38 | 1.1 | 14.27 |
| 640.16 | 83.35 | 1.16 | 14.27 |
| 647.27 | 88.96 | 1.03 | 14.35 |
| 647.27 | 85.23 | 1.12 | 14.35 |
| 654.39 | 92.64 | 0.96 | 14.43 |
| 654.39 | 84.9 | 1.14 | 14.43 |
| 654.39 | 86.2 | 1.11 | 14.43 |
| 661.5 | 87.37 | 1.09 | 14.51 |
| 661.5 | 88.65 | 1.06 | 14.51 |
| 661.5 | 85.69 | 1.13 | 14.51 |
| 668.61 | 87.48 | 1.1 | 14.59 |
| 668.61 | 85.38 | 1.15 | 14.59 |
| 668.61 | 92.84 | 0.97 | 14.59 |
| 668.61 | 92.22 | 0.99 | 14.59 |
| 668.61 | 85.38 | 1.15 | 14.59 |
| 675.72 | 86.13 | 1.14 | 14.67 |
| 675.72 | 93.63 | 0.97 | 14.67 |
| 675.72 | 90.43 | 1.04 | 14.67 |
| 675.72 | 88.89 | 1.07 | 14.67 |
| 689.95 | 89.46 | 1.08 | 14.82 |
| 689.95 | 89.15 | 1.09 | 14.82 |
| 689.95 | 89.13 | 1.09 | 14.82 |
| 697.06 | 98.68 | 0.9 | 14.9 |
| 704.18 | 92.9 | 1.03 | 14.97 |
| 704.18 | 93.94 | 1 | 14.97 |
| 711.29 | 91.69 | 1.06 | 15.05 |
| 711.29 | 90.37 | 1.09 | 15.05 |
| 711.29 | 94.49 | 1 | 15.05 |
| 711.29 | 105.19 | 0.81 | 15.05 |
| 718.4 | 93.39 | 1.04 | 15.12 |
| 718.4 | 90.41 | 1.1 | 15.12 |
| 718.4 | 91.18 | 1.09 | 15.12 |
| 718.4 | 92.4 | 1.06 | 15.12 |
| 718.4 | 93.45 | 1.03 | 15.12 |
| 725.51 | 90.43 | 1.11 | 15.2 |
| 739.74 | 94.62 | 1.04 | 15.34 |
| 753.97 | 100.16 | 0.94 | 15.49 |
| 753.97 | 108.14 | 0.81 | 15.49 |
| 761.08 | 98.17 | 0.99 | 15.56 |
| 768.19 | 91.78 | 1.15 | 15.64 |
| 775.3 | 96.45 | 1.05 | 15.71 |
| 775.3 | 95.66 | 1.06 | 15.71 |
| 775.3 | 103.16 | 0.92 | 15.71 |
| 789.53 | 96.87 | 1.06 | 15.85 |
| 789.53 | 104.95 | 0.9 | 15.85 |
| 789.53 | 94.12 | 1.12 | 15.85 |
| 796.64 | 110.44 | 0.82 | 15.92 |
| 796.64 | 105.08 | 0.91 | 15.92 |
| 817.98 | 97.14 | 1.09 | 16.14 |
| 825.1 | 106.01 | 0.92 | 16.21 |
| 825.1 | 110.9 | 0.84 | 16.21 |
| 825.1 | 100.2 | 1.03 | 16.21 |
| 825.1 | 99.61 | 1.05 | 16.21 |
| 832.21 | 100.89 | 1.03 | 16.28 |
| 839.32 | 99.36 | 1.07 | 16.35 |
| 839.32 | 97.64 | 1.11 | 16.35 |
| 853.55 | 99.92 | 1.07 | 16.48 |
| 867.77 | 103.91 | 1.01 | 16.62 |
| 889.11 | 102.7 | 1.06 | 16.82 |
| 889.11 | 100.71 | 1.1 | 16.82 |
| 896.22 | 113.13 | 0.88 | 16.89 |
| 896.22 | 113.44 | 0.88 | 16.89 |
| 917.56 | 129.01 | 0.69 | 17.09 |
| 924.68 | 102.8 | 1.1 | 17.16 |
| 931.79 | 119.52 | 0.82 | 17.22 |
| 931.79 | 106.42 | 1.03 | 17.22 |
| 938.9 | 109.07 | 0.99 | 17.29 |
| 938.9 | 104.83 | 1.07 | 17.29 |
| 946.01 | 102.06 | 1.14 | 17.35 |
| 960.24 | 108.34 | 1.03 | 17.48 |
| 974.47 | 105.43 | 1.1 | 17.61 |
| 974.47 | 113.5 | 0.95 | 17.61 |
| 974.47 | 106.11 | 1.09 | 17.61 |
| 981.58 | 107.39 | 1.07 | 17.68 |
| 981.58 | 113.88 | 0.95 | 17.68 |
| 988.69 | 111.23 | 1 | 17.74 |
| 1002.92 | 131.88 | 0.72 | 17.87 |
| 1002.92 | 111.19 | 1.02 | 17.87 |
| 1010.03 | 111.28 | 1.03 | 17.93 |
| 1010.03 | 123.58 | 0.83 | 17.93 |
| 1017.14 | 111.63 | 1.03 | 17.99 |
| 1024.26 | 115.45 | 0.97 | 18.06 |
| 1024.26 | 108.89 | 1.09 | 18.06 |
| 1038.48 | 110.92 | 1.06 | 18.18 |
| 1038.48 | 119.68 | 0.91 | 18.18 |
| 1045.59 | 111.28 | 1.06 | 18.24 |
| 1045.59 | 118.95 | 0.93 | 18.24 |
| 1045.59 | 149.19 | 0.59 | 18.24 |
| 1045.59 | 111.14 | 1.06 | 18.24 |
| 1052.71 | 114.87 | 1 | 18.31 |
| 1059.82 | 111.54 | 1.07 | 18.37 |
| 1066.93 | 113.46 | 1.04 | 18.43 |
| 1074.05 | 112.58 | 1.06 | 18.49 |
| 1081.16 | 116.22 | 1.01 | 18.55 |
| 1088.27 | 116.15 | 1.01 | 18.61 |
| 1109.61 | 114.38 | 1.07 | 18.79 |
| 1152.29 | 117.36 | 1.05 | 19.15 |
| 1152.29 | 116.88 | 1.06 | 19.15 |
| 1173.63 | 123.91 | 0.96 | 19.33 |
| 1173.63 | 127.11 | 0.91 | 19.33 |
| 1180.74 | 115.89 | 1.1 | 19.39 |
| 1180.74 | 118.16 | 1.06 | 19.39 |
| 1187.85 | 127.6 | 0.92 | 19.44 |
| 1194.97 | 120.12 | 1.04 | 19.5 |
| 1230.53 | 125.37 | 0.98 | 19.79 |
| 1237.64 | 127.84 | 0.95 | 19.85 |
| 1244.76 | 124.42 | 1.01 | 19.91 |
| 1266.09 | 126.3 | 1 | 20.08 |
| 1301.66 | 124.88 | 1.05 | 20.36 |
| 1308.77 | 123.89 | 1.07 | 20.41 |
| 1315.88 | 128.9 | 1 | 20.47 |
| 1323 | 129.12 | 1 | 20.52 |
| 1337.22 | 133.18 | 0.95 | 20.63 |
| 1365.67 | 133 | 0.97 | 20.85 |
| 1372.79 | 147.65 | 0.79 | 20.9 |
| 1387.01 | 133.55 | 0.98 | 21.01 |
| 1394.13 | 158.85 | 0.69 | 21.07 |
| 1394.13 | 136.64 | 0.94 | 21.07 |
| 1408.35 | 133.31 | 1 | 21.17 |
| 1436.8 | 132.58 | 1.03 | 21.39 |
| 1500.82 | 142.11 | 0.93 | 21.86 |
| 1507.93 | 136.64 | 1.01 | 21.91 |
| 1515.05 | 136.11 | 1.03 | 21.96 |
| 1529.27 | 142.97 | 0.94 | 22.06 |
| 1557.72 | 142.11 | 0.97 | 22.27 |
| 1564.84 | 136.07 | 1.06 | 22.32 |
| 1628.85 | 157.31 | 0.83 | 22.77 |
| 1643.08 | 154.9 | 0.86 | 22.87 |
| 1650.19 | 141.36 | 1.04 | 22.92 |
| 1671.53 | 151.82 | 0.91 | 23.07 |
| 1685.75 | 166.15 | 0.77 | 23.16 |
| 1728.43 | 163.57 | 0.81 | 23.46 |
| 1764 | 153.73 | 0.94 | 23.7 |
| 1778.22 | 153.6 | 0.95 | 23.79 |
| 1785.34 | 146.61 | 1.04 | 23.84 |
| 1835.13 | 152.88 | 0.99 | 24.17 |
| 1856.46 | 156.07 | 0.96 | 24.31 |
| 1856.46 | 189.53 | 0.65 | 24.31 |
| 1906.25 | 162.53 | 0.91 | 24.63 |
| 1913.37 | 187.55 | 0.68 | 24.68 |
| 1934.71 | 157.33 | 0.98 | 24.82 |
| 1956.04 | 163.68 | 0.92 | 24.95 |
| 1956.04 | 157.79 | 0.99 | 24.95 |
| 2119.64 | 183.58 | 0.79 | 25.98 |
| 2155.21 | 174.84 | 0.89 | 26.19 |
| 2176.54 | 177.84 | 0.86 | 26.32 |
| 2325.91 | 179.96 | 0.9 | 27.21 |
| 2333.03 | 175.88 | 0.95 | 27.25 |
| 2461.06 | 183.36 | 0.92 | 27.99 |
| 2546.41 | 186.11 | 0.92 | 28.47 |
| 2567.75 | 191.01 | 0.88 | 28.59 |
| 2574.87 | 191.19 | 0.89 | 28.63 |
| 2610.43 | 192.91 | 0.88 | 28.83 |
| 2660.22 | 230.82 | 0.63 | 29.1 |
| 2688.67 | 183.82 | 1 | 29.25 |
| 2930.51 | 224.71 | 0.73 | 30.54 |
| 2966.07 | 198.58 | 0.95 | 30.73 |
| 3058.54 | 198.69 | 0.97 | 31.2 |
| 3108.33 | 209.8 | 0.89 | 31.45 |
| 3321.72 | 217.9 | 0.88 | 32.52 |
| 3371.51 | 211.81 | 0.94 | 32.76 |
| 3769.83 | 242.51 | 0.81 | 34.64 |
| 3869.41 | 237.22 | 0.86 | 35.1 |
| 4153.93 | 245.38 | 0.87 | 36.36 |
| 4673.17 | 253.12 | 0.92 | 38.57 |
| 4680.28 | 272.91 | 0.79 | 38.6 |
| 4772.75 | 263.66 | 0.86 | 38.98 |
| 5604.96 | 277.38 | 0.92 | 42.24 |
| 5868.13 | 280.01 | 0.94 | 43.22 |
| 6486.95 | 335.28 | 0.73 | 45.44 |
| 7304.94 | 309.81 | 0.96 | 48.22 |
| 7361.84 | 320.33 | 0.9 | 48.41 |
| 8670.61 | 364.13 | 0.82 | 52.54 |
| 9040.48 | 385.73 | 0.76 | 53.64 |
| 9253.87 | 378.47 | 0.81 | 54.27 |
| 9389.01 | 387.01 | 0.79 | 54.67 |

SI Table 3: The area, perimeter, circularity and average radii of all the aggregates in the in the images obtained at 48 hours.

| **Area (µm^2^)** | **Perimeter (µm)** | **Circularity** | **Average Radii (µm)** |
| --- | --- | --- | --- |
| 184.94 | 49.72 | 0.94 | 7.67 |
| 220.5 | 52.67 | 1 | 8.38 |
| 248.95 | 66.43 | 0.71 | 8.9 |
| 277.4 | 60.96 | 0.94 | 9.4 |
| 305.85 | 89.44 | 0.48 | 9.87 |
| 320.08 | 72.9 | 0.76 | 10.09 |
| 320.08 | 67.67 | 0.88 | 10.09 |
| 369.87 | 78.94 | 0.75 | 10.85 |
| 384.1 | 98.92 | 0.49 | 11.06 |
| 419.66 | 96.89 | 0.56 | 11.56 |
| 540.58 | 89.46 | 0.85 | 13.12 |
| 611.71 | 107.53 | 0.66 | 13.95 |
| 668.61 | 94.38 | 0.94 | 14.59 |
| 689.95 | 132.1 | 0.5 | 14.82 |
| 739.74 | 132.03 | 0.53 | 15.34 |
| 796.64 | 108.58 | 0.85 | 15.92 |
| 874.89 | 115.91 | 0.82 | 16.69 |
| 946.01 | 121.88 | 0.8 | 17.35 |
| 1017.14 | 185.59 | 0.37 | 17.99 |
| 1081.16 | 121.07 | 0.93 | 18.55 |
| 1166.51 | 155.78 | 0.6 | 19.27 |
| 1401.24 | 204.55 | 0.42 | 21.12 |
| 1401.24 | 151.59 | 0.77 | 21.12 |
| 1635.96 | 182.1 | 0.62 | 22.82 |
| 1643.08 | 160.13 | 0.81 | 22.87 |
| 1721.32 | 173.54 | 0.72 | 23.41 |
| 1856.46 | 229.72 | 0.44 | 24.31 |
| 1856.46 | 192.75 | 0.63 | 24.31 |
| 1856.46 | 187.13 | 0.67 | 24.31 |
| 2076.96 | 174.05 | 0.86 | 25.71 |
| 2105.42 | 190.94 | 0.73 | 25.89 |
| 2126.75 | 180.8 | 0.82 | 26.02 |
| 2162.32 | 196.33 | 0.7 | 26.24 |
| 2176.54 | 192.42 | 0.74 | 26.32 |
| 2261.9 | 193.55 | 0.76 | 26.83 |
| 2290.35 | 190.09 | 0.8 | 27 |
| 2418.38 | 180.87 | 0.93 | 27.75 |
| 2731.35 | 260.75 | 0.5 | 29.49 |
| 2788.25 | 203.32 | 0.85 | 29.79 |
| 2873.61 | 217.65 | 0.76 | 30.24 |
| 3250.59 | 219.24 | 0.85 | 32.17 |
| 3456.86 | 250.01 | 0.69 | 33.17 |
| 3883.64 | 238.19 | 0.86 | 35.16 |
| 4004.56 | 286.01 | 0.62 | 35.7 |
| 4068.57 | 246.88 | 0.84 | 35.99 |
| 4068.57 | 248.49 | 0.83 | 35.99 |
| 4402.88 | 259.85 | 0.82 | 37.44 |
| 4502.46 | 243.57 | 0.95 | 37.86 |
| 4680.28 | 269 | 0.81 | 38.6 |
| 4815.43 | 307.62 | 0.64 | 39.15 |
| 4865.22 | 256.7 | 0.93 | 39.35 |
| 5185.3 | 274.45 | 0.87 | 40.63 |
| 5235.09 | 271.41 | 0.89 | 40.82 |
| 5548.05 | 279.48 | 0.89 | 42.02 |
| 5668.97 | 276.94 | 0.93 | 42.48 |
| 5690.31 | 313.45 | 0.73 | 42.56 |
| 5747.21 | 283.23 | 0.9 | 42.77 |
| 5903.7 | 300.63 | 0.82 | 43.35 |
| 7354.73 | 325.86 | 0.87 | 48.38 |
| 7475.65 | 331.4 | 0.86 | 48.78 |
| 7546.78 | 546.96 | 0.32 | 49.01 |
| 7724.6 | 355.51 | 0.77 | 49.59 |
| 7767.27 | 345.38 | 0.82 | 49.72 |
| 8115.81 | 344.66 | 0.86 | 50.83 |
| 8165.6 | 349.22 | 0.84 | 50.98 |
| 8350.53 | 360.54 | 0.81 | 51.56 |
| 8606.6 | 418.72 | 0.62 | 52.34 |
| 8613.71 | 358.91 | 0.84 | 52.36 |
| 9723.32 | 367.16 | 0.91 | 55.63 |
| 9901.14 | 462.92 | 0.58 | 56.14 |
| 10989.41 | 525.01 | 0.5 | 59.14 |
| 11266.82 | 540.48 | 0.48 | 59.89 |
| 11387.74 | 448.57 | 0.71 | 60.21 |
| 11551.33 | 406.17 | 0.88 | 60.64 |
| 13009.47 | 506.49 | 0.64 | 64.35 |
| 13101.94 | 421.86 | 0.93 | 64.58 |
| 13350.89 | 493.94 | 0.69 | 65.19 |
| 13585.62 | 475.17 | 0.76 | 65.76 |
| 14346.7 | 454.92 | 0.87 | 67.58 |
| 15627.02 | 518.42 | 0.73 | 70.53 |
| 17120.72 | 502.19 | 0.85 | 73.82 |
| 17753.77 | 512.33 | 0.85 | 75.17 |
| 19176.35 | 566.28 | 0.75 | 78.13 |
| 19247.48 | 537.46 | 0.84 | 78.27 |
| 19980.11 | 782.08 | 0.41 | 79.75 |
| 22832.37 | 586.64 | 0.83 | 85.25 |
| 23586.34 | 622.06 | 0.77 | 86.65 |
| 23657.47 | 606.12 | 0.81 | 86.78 |
| 29817.23 | 663.82 | 0.85 | 97.42 |
| 31652.36 | 855.69 | 0.54 | 100.38 |
| 31787.5 | 700.92 | 0.81 | 100.59 |
| 33473.26 | 752.31 | 0.74 | 103.22 |
| 33515.93 | 775.36 | 0.7 | 103.29 |
| 34042.29 | 716.95 | 0.83 | 104.1 |
| 35607.12 | 742.3 | 0.81 | 106.46 |
| 36019.67 | 901.26 | 0.56 | 107.08 |
| 36297.07 | 780.32 | 0.75 | 107.49 |
| 39881.97 | 848.92 | 0.7 | 112.67 |
| 41212.08 | 807.34 | 0.79 | 114.53 |
| 41482.37 | 815.7 | 0.78 | 114.91 |

**SI Table 4:** Various techniques and the drawbacks associated with them for analyzing aggregates in IgG sample in serum (Filipe et al., 2012; Demeule et al., 2009; Zölls et al., 2012).

| **Technique** | **Drawback** |
| --- | --- |
| Fluorescence Single Particle Tracking | Size measurement upto 1 μm |
| Flow Cytometry | Significant optimization of instrument settings, sample dilution |
| SEC | Sample dilution |
| Field Flow Fractionation | Sample dilution |
| Analytical ultracentrifugation with fluorescence | Application of centrifugal force |

**References**

1. Filipe, V., Poole, R., Oladunjoye, O., Braeckmans, K. and Jiskoot, W., 2012. Detection and characterization of subvisible aggregates of monoclonal IgG in serum. *Pharmaceutical research*, *29*(8), 2202-2212.
2. Demeule, B., Shire, S.J. and Liu, J., 2009. A therapeutic antibody and its antigen form different complexes in serum than in phosphate-buffered saline: a study by analytical ultracentrifugation. *Analytical biochemistry*, *388*(2), 279-287.
3. Zölls, S., Tantipolphan, R., Wiggenhorn, M., Winter, G., Jiskoot, W., Friess, W. and Hawe, A., 2012. Particles in therapeutic protein formulations, Part 1: Overview of analytical methods. *Journal of pharmaceutical sciences*, *101*(3), 914-935.
